# From Metaphors to Formalism: A Heuristic Approach to Holistic Assessments of Ecosystem Health

**DOI:** 10.1371/journal.pone.0159481

**Published:** 2016-08-10

**Authors:** Heino O. Fock, Gerd Kraus

**Affiliations:** Thünen Institute of Sea Fisheries, Hamburg, Germany; US Army Engineer Research and Development Center, UNITED STATES

## Abstract

Environmental policies employ metaphoric objectives such as ecosystem health, resilience and sustainable provision of ecosystem services, which influence corresponding sustainability assessments by means of normative settings such as assumptions on system description, indicator selection, aggregation of information and target setting. A heuristic approach is developed for sustainability assessments to avoid ambiguity and applications to the EU Marine Strategy Framework Directive (MSFD) and OSPAR assessments are presented. For MSFD, nineteen different assessment procedures have been proposed, but at present no agreed assessment procedure is available. The heuristic assessment framework is a functional-holistic approach comprising an ex-ante/ex-post assessment framework with specifically defined normative and systemic dimensions (EAEPNS). The outer normative dimension defines the ex-ante/ex-post framework, of which the latter branch delivers one measure of ecosystem health based on indicators and the former allows to account for the multi-dimensional nature of sustainability (social, economic, ecological) in terms of modeling approaches. For MSFD, the ex-ante/ex-post framework replaces the current distinction between assessments based on pressure and state descriptors. The ex-ante and the ex-post branch each comprise an inner normative and a systemic dimension. The inner normative dimension in the ex-post branch considers additive utility models and likelihood functions to standardize variables normalized with Bayesian modeling. Likelihood functions allow precautionary target setting. The ex-post systemic dimension considers *a posteriori* indicator selection by means of analysis of indicator space to avoid redundant indicator information as opposed to *a priori* indicator selection in deconstructive-structural approaches. Indicator information is expressed in terms of ecosystem variability by means of multivariate analysis procedures. The application to the OSPAR assessment for the southern North Sea showed, that with the selected 36 indicators 48% of ecosystem variability could be explained. Tools for the ex-ante branch are risk and ecosystem models with the capability to analyze trade-offs, generating model output for each of the pressure chains to allow for a phasing-out of human pressures. The Bayesian measure of ecosystem health is sensitive to trends in environmental features, but robust to ecosystem variability in line with state space models. The combination of the ex-ante and ex-post branch is essential to evaluate ecosystem resilience and to adopt adaptive management. Based on requirements of the heuristic approach, three possible developments of this concept can be envisioned, i.e. a governance driven approach built upon participatory processes, a science driven functional-holistic approach requiring extensive monitoring to analyze complete ecosystem variability, and an approach with emphasis on ex-ante modeling and ex-post assessment of well-studied subsystems.

## Introduction

“The validity of a conclusion may be regarded as a compound event, depending upon the premises happening to be true; thus, to obtain the probability of the conclusion, we must multiply together the fractions expressing the probabilities of the premises.” W. S. Jevons, The Principles of Science, 1877, p. 209

Lakoff and Johnson [1] argue that human cognitive concepts are all embedded in metaphors determining thought, language and action. Metaphors are either descriptive (size, orientation) or map complex structures onto simpler objects, and are therefore easy to communicate and aspirational, figurative or iconic [1,2]. The understanding of metaphors depends on the cultural context and its societal value framework within a ‘metaphoric web‘, but also on subjective understanding [3–5].

Environmental policies such as the 1992 Rio Declaration on Environment and Development (Principle 7), the Millennium Ecosystem Assessment [6] and the Sustainability Development Goals as follow-up (e.g. [7]), the EU Marine Strategy Framework Directive (MSFD, 2008/56/EC, Art 3(5)) and the US National Ocean Policy (Executive Order 13547 on July 19th, 2010) commonly employ a range of ecological metaphors to convey their valued objectives and goals: inter alia ‘healthy environment’, ‘blue wealth, ‘productive ecosystems’, ‘ecosystem services’. ‘Ecosystem health’ appears as anoverarching principle indicative of unimpaired ecosystem functioning and sustainable use of ecosystem services [8–11]. ‘Ecosystem health’ is considered integrative and trans-disciplinary in that it combines the knowledge of the ecosystem with the knowledge of what is desirable, which necessitates consideration of all determinants of societal values, e.g. economic and cultural opportunities and human health [12,13].

## Rationale and outline of article

Environmental metaphors express complex concepts (‘principal subject’ [14]) by means of simple analogous sets of known entities (‘subsidiary subject’ [14]). This replacement is not possible without change of cognitive contents of the two subjects [14], and thus metaphors represent a certain cognitive strategy of analogical problem solving [1,15]. As such, Moser [15] interprets metaphors as tacit and *a priori* knowledge. Mikkelson [16] sees a danger in applying definitions that turn an empirical matter regarded as important into a generic *a priori* exercise. A single observation of ecosystem disorder would be such an empirical matter, but its generalization would then place it inside the metaphoric web of ecosystem health and thus establish it as part of the *a priori* tacit knowledge around this subject. Black [14] refers to this replacement process as ‘interaction’ (i.e. interaction metaphors, ‘conceptual metaphors’ [5] or 'structural metaphors' [1]), and distinguishes between ‘interaction views’ in the application of metaphors as opposed to ‘substitution’ and ‘comparison views’ with a 1:1 replacement in meaning (‘strong as a lion’). The transition from ‘comparative’ to ‘interaction view’ is gradual, since each metaphor comprises both descriptive and evaluative elements ('lion' comprises also a value) [5]. Evaluative elements inherently contain reflections on goals (‘is/ought problem‘, [5,17]), and metaphors with predominantly evaluative character are termed ‘generative metaphors’ according to Schön [18]. Schön identified three stages in the replacement process to support policy making: ‘Generative metaphors’ provide or restructure a decision making framework (stage 2) for problem solving (stage 3) after problems have been identified in a first stage [18]. Thus, at the second step metaphors have a heuristic and stimulating value expressing something that could not be expressed otherwise but are not yet operational [5,19–21]. From step 2 to step 3, generative metaphors require additional interpretation and–multiple–transformations within the metaphoric web to become a policy instrument in an operational context [14]. Examples from the OSPAR convention area and the Great Lakes show that the term ecosystem health was adopted after negative observations in certain environmental sectors (e.g. eutrophication, pollution as indicators of ecosystem distress, step 1) had led to the term (and metaphor) of ecosystem approach to management, which was transformed into the concept of ecosystem health as policy tool (step 2) operationalized in the first place by a suite of performance indicators (step 3) [22,23].

Two implications arise from this translation process: Scientific analysis may lose its specificity and rigor, because scientific assertions are made that are not entirely based on data but values [18,24–27]. This constitutes 'normative science', and in this context Sarewitz speaks of 'scientization' of political disputes ([4], and references in [28]). Secondly, metaphors act as selection filters for the understanding of systems (for ecosystem services see discussion in Menzie et al. [29]), channel the flow of information in scientific analyses [27,30] and influence methodologies and analytical concepts [2,31].

In line with these arguments, we reverse Rapport's claim of being explicit about the dual character of metaphors in political debate comprising scientific facts and policy goals ([12], p. 42), and in turn postulate that metaphors of ecosystem health have a strong influence on the development of environmental assessments where this explicit differentiation has not been achieved. We argue that it is possible to develop a formalistic heuristic approach to functional-holistic assessments of ecosystem health, minimizing metaphorical influence and separating value system and scientific methods. The method applied is reconstructive metaphor analysis [32,33], i.e. to determine metaphor use in relation to an external reference system, which here is the use of metaphors in the context of sustainability assessment methodologies. Lakoff and Johnson term this ‘grounding a metaphor’ in a framework which can be directly understood ([1], p. 57), and Black defines this as a process of certification of meaning, which allows one to check a metaphor against its necessary associated statement ([19], p. 64). The latter may contain axiomatic statements like mathematical expressions and logical statements. Since metaphors possess no ‘truth condition’ as opposite to falsifiable scientific theories, the question of metaphor use is not whether they are wrong but where they are misleading [19–21]. The linkage between sustainability and ecosystem health [8,34,35] allows us to adopt the methodology of sustainability assessments to assessments of ecosystem health. After reviewing the metaphor ‘ecosystem health’, we will provide an overview of sustainability assessment methodologies to obtain building blocks for the heuristic approach: the model of Binder et al. [36], the ex-post/ex-ante framework, indicator selection and indicator aggregation methodologies. The heuristic approach is outlined in the third section, in the first place as tailor-made approach for the MSFD, given that MSFD to date has no operational assessment protocol. The fourth section considers comparing the heuristic approach to environmental policies such as HOLAS for the Baltic Sea [37], OSPAR indicator based ecosystem assessments [38], for which an example is calculated, and EU Water Framework Directive (2000/60/EC; see [39]). These policy applications are discussed against the background of existing scientific literature on ecosystem health assessments.

## Ecosystem health as metaphor

Ecosystem health is a two-stage metaphor with regards to the terms 'ecosystem' and 'health'. As a transformation of the concept of human health it has helped to facilitate a better understanding of ecosystem state for management, providing a language to describe dysfunctions and illness of ecosystems [13,23,24,40,41]. In this negative definition, health thus means being free of disease and distress (in [42]). This way of understanding is linked to a background where ecosystems can develop their full potential in the absence of serious anthropogenic perturbations and cumulative impacts [40,43,44]. Accordingly, Calow [45] distinguishes between weak and strong analogies of health, with strong analogies making profound assumptions on system dynamics such as homeostatic processes and equilibrium states, whereas weak analogies simply state deviations from normality. The provisioning of ecosystem services appears often as an intrinsic component of the positive understanding of the term 'health' describing "the system's ability to realize functions desired by society and to maintain structures needed by its functions and by society over a long time" [42]. However, the application of ecosystem services to assess ecosystem health is problematic, since it remains unclear how changes in ecosystem services will affect changes of ecosystem health [9], and how this depends on the selection of ecosystem services to describe ecosystem health [29].

The system's part of the metaphor either understands health as an emergent property of the entire system as a unit, with the possibility to assess overall ecosystem health as part of system's integrity [9,13], or as a characteristic of the parts of the system evidenced through individual indicators [46,47], of which ecosystem services are regarded as one type of indicator [13]. Emergent properties are linked to the capabilities of a system to self-organize [48,49]. However, opposite to homeostasis in organisms, ecosystems have completely different control systems based on non-mutualistic mechanisms such as competition or predation [45]. In fact, the prevalence of communality as a principle of ecosystem organization constitutes a significant misunderstanding of ecosystem functioning [50].

For assessments of ecosystem health, Borja et al. [51] distinguish between a deconstructive-structural assessment type based on compartmentalized ecosystem information in terms of indicators (the whole is the sum of parts as metaphor, see Table 1), and a functional-holistic approach assessing ecosystem health as a whole (metaphor of ecosystems as meta-organisms [8,9,23], but see [9,13]). Both approaches imply that recombining a set of characteristic features provides an accurate representation of ecosystem functioning, but the deconstructive-structural method is characterized by significant *a priori* assumptions about the functioning of the system [51]. As for the selection of ecosystem services, the sum-of-parts metaphor may lead to considerable confusion in selecting indicators and metrics, in particular in cases where guidance warrants the analysis of “the essential features and characteristics" [52].

**Table 1:** Examples of interaction metaphors with regards to ecosystem health in relation to assessment type. Sources: [2,5,26,48,63–67]

Interaction metaphors associate d with. . .^a^	
. . . deconstructive- structural assessment type	Ecosystem as suite of components
	Ecosystem services
. . . functional- holistic assessment type	Ecosystem as meta-organism
	Emergence (self-organization)
	Ascendency (self-organization)
	Gaia theory ^b^
. . . both	Ecosystem health
	Ecosystem approach
	Resilience
	Tipping points (early warning)
	Vulnerability
	Vitality of socio-ecological systems
. . . assessment methods	Hierarchy, hierarchical designs (pyramid, spider webs)
	Everything must be in healthy condition (one-out-all-out)
	Keystone, flagship species

^a^ Fischherz [65] lists 494 metaphors for sustainability, of which 36 originated from an ecological and 336 from a sociological context. 24 metaphors were directly linked to ecosystem health. In turn, Larson [5] lists ecosystem health under the topic conservation biology, together with 19 other metaphors in this category.

^b^ Larson [5] links the origin of holistic approaches to the Gaia theory.

**Fig 1:**
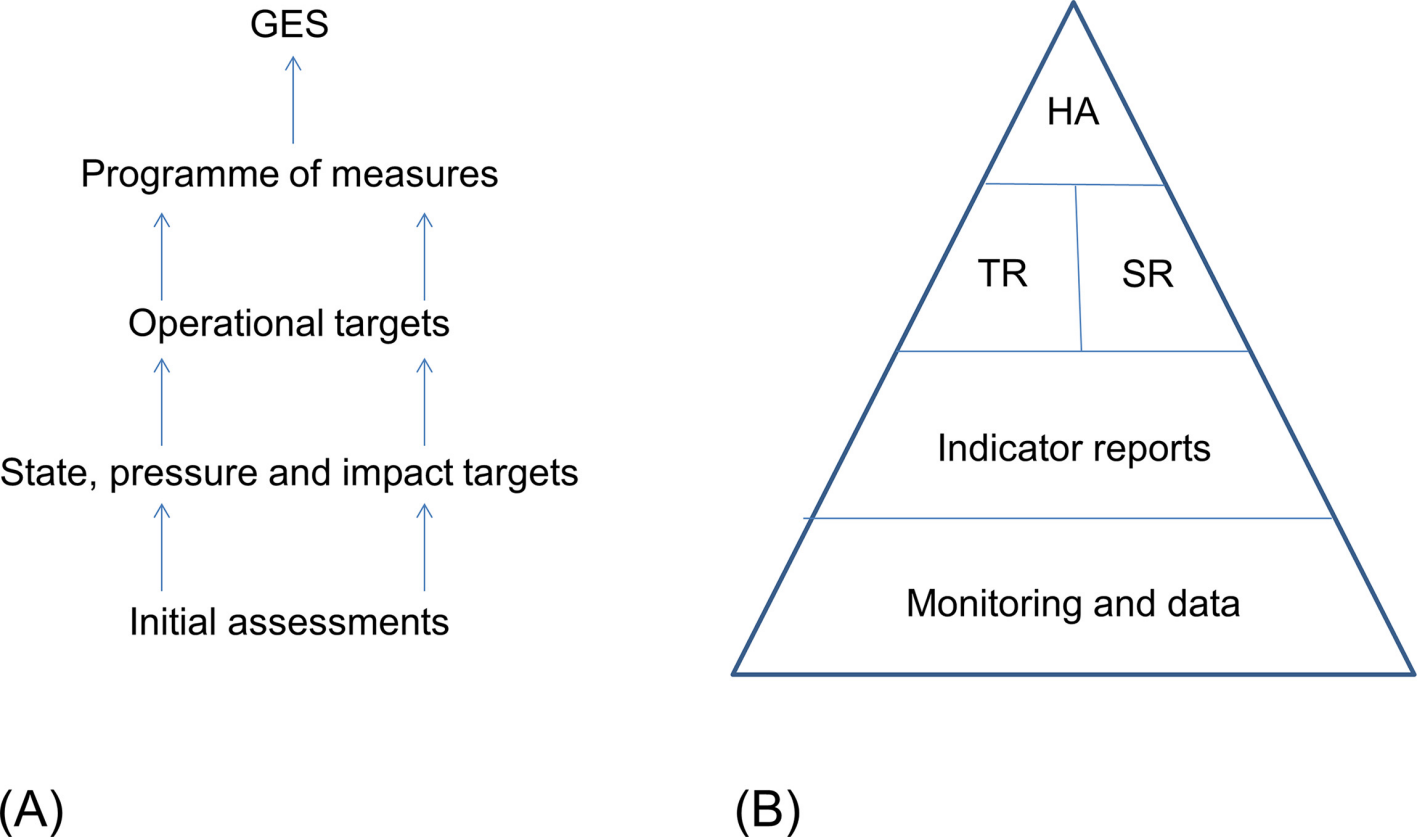
Deconstructing-structural hierarchical metaphors of ecosystem health. (A) Scheme applied in the EU Marine Strategy Framework Directive (after [59]); (B) Scheme applied Holistic Assessment (HOLAS) of the Baltic Sea (after [37]). GES–good environmental status, HA–holistic assessment, SR–scientific reports, TR–Thematic reports

Deconstructing-structural assessment types either apply linear or hierarchical schemes. Linear schemes at indicator level likely indicate how deconstructing-structural approaches have developed: Jörgensen et al. [53] describe this for ecosystem health assessments, starting with single observations and the question: "What is wrong?", followed by a subsequent management measure, so that a reference to the system level is not needed. In practice, most concepts are deconstructive-structural (see [54]), with a hierarchical aggregation of information (top of pyramid as metaphor) retrieved from key indicators or key functions (key role as functional metaphor) (Fig 1). At the regional scale in marine assessments, Helsinki Commission (HELCOM) assessments for the Baltic Sea region (HOLAS) aggregate information from 3 thematic assessments for contaminants, biodiversity and eutrophication condensed into one measure of ecosystem health accompanied by analyses of human pressures [37]. Oslo-Paris Commission (OSPAR) assessments for the North-east Atlantic apply 9 thematic assessments that are qualitatively aggregated into regional summaries [55]. The MSFD applies a hierarchy of attributes, indicators, criteria and descriptors to assess ecosystem health. In the Great Lakes region, as part of the NOAA goals for healthy oceans and coastal communities (http://www.ppi.noaa.gov/goals/) aligned to the US National Ocean Policy, ecosystem health is assessed by means of 53 indicator reports aggregated into reports on physical, chemical and biological integrity [56]. All aforementioned deconstructive-structural approaches lack guidance to achieve the overall assessment (see for Great Lakes [57], HOLAS [37,58], MSFD [59–61], OSPAR [55] despite some progress during the ‘Utrecht workshop’ [62]).

Functional-holistic assessments focus on the analysis of the entire ecosystem, which is expressed by means of emergent properties such as energy (e.g. exergy), network complexity (e.g. entropy, ascendency) [35,44,47], homeostasis, balance between system components, and the constituting criteria for ecosystem health, i.e. system vigor, resilience and diversity [26,40,43]. This in part reflects the influence of the metaphoric web, since vigor, resilience and diversity themselves are metaphors, which in turn are further described along dimensions termed brittle, eutrophic and crystallized [8].

## Sustainability assessment methodology

### Dimensions of sustainability assessments: the Binder et al. model

Sustainability assessments provide decision-makers with evaluations of social-ecological systems within a multi-dimensional context to support decision making [68,69]: ecological, economic, social (i.e. triple baseline; further spatial and human health dimensions according to Rapport [70] can be assigned to the former three). Sustainability assessments comprise four generic features: consideration of equity (among people, species, generations, geographic regions), adopting a holistic perspective (view the entire system, integrate across sectors and disciplines), incorporation of the triple base-line into the decision-making process (i.e. normative settings, participatory processes, methodology and treatment of uncertainties and risks, adoption of the precautionary approach), and support of decision-making (governance issues, communicative processes)[69,71]. Two different lines of assessment approaches have evolved with regards to the treatment of the multi-dimensional context: firstly, integrated social, economic and ecological sustainability assessments in terrestrial and marine ecosystems [6,72,73], and secondly ecosystem-based assessments, mainly applied to marine systems, with subsequent consideration of the socio-economic context [74,75].

**Fig 2:**
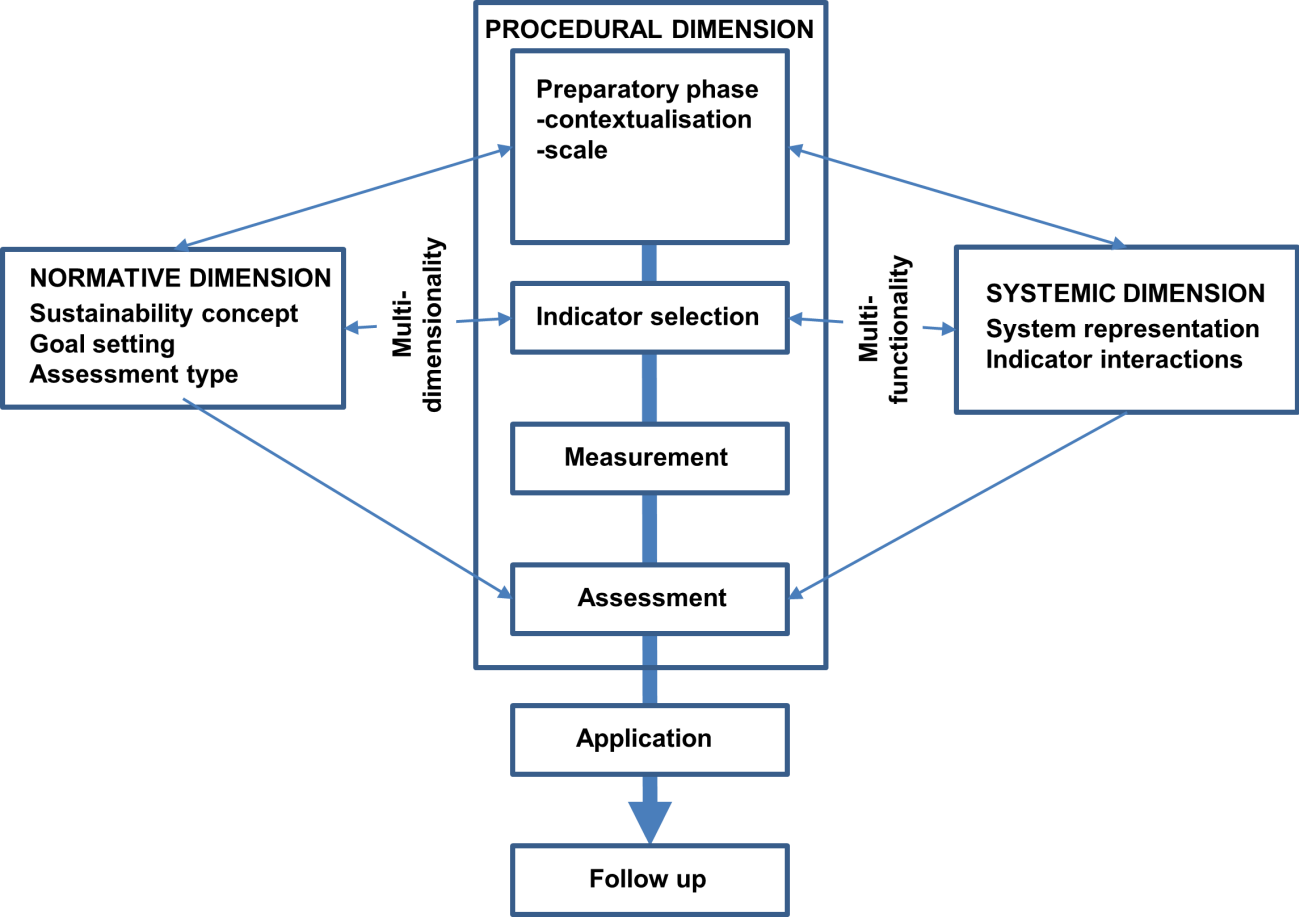
Dimensions and flow chart of sustainability assessments modified after Binder et al. [36].

Binder et al. [36,76] described a workflow to coordinate the assessment features into three interacting assessment dimensions, i.e. normative, systemic and procedural (Fig 2), of which the procedural dimension can be considered as umbrella for the normative dimension defining the boundary conditions, and the systemic dimension making the assessment operational. The normative dimension transfers political preferences and value-based assumptions into an assessment framework: It defines the sustainability concept and the choice between weak and strong sustainability [77]. It comprises a formulation of target setting [78], a definition of the assessment type, assessment method and method of aggregation of assessment information [79]. The normative dimension further prescribes how those social-ecological components identified during a scoping phase interact within and between the normative and systemic dimensions and thus how to integrate multidimensionality [36,75,80](Fig 2).

### The ex-ante/ex-post framework

Assessment method can be described in terms of the ex-ante/ex-post methodologies. Ex-ante assessments based on modeling allow for a prospective look on the social-ecological system and the assessment of alternatives. In turn, ex-post assessments are based on existing data and put an emphasis on retrospective evaluation [54,69,71]. Accordingly, Ness et al. [69] distinguish between integrated or model based and indicator based assessments for ecosystems, but add product-related indices with emphasis on the production process as third category. It is widely accepted that neither applying indicators in an ex-post approach [81,82] nor applying ex-ante modelling [83] let alone are not sufficient for a holistic assessment, but that a combination of ex-ante and ex-post methods is essential for adaptive management designs, revealed through explicit modelling and monitoring steps [75,84].The ex-ante/ex-post distinction is needed for evaluating the resilience in the dynamics of social-ecological systems as sustainability assessment criterion [85,86].

### Indicator selection in the systemic dimension

The systemic dimension describes the system in quantitative terms in a way that allows for detection of change towards the sustainability goal (see [36]). System description is considered *a priori* if the interpretation framework is existing before the analysis takes place and is typical of deconstructive-structural assessments ('top-down' sensu [79]). This hampers the flow of information and thus can create bias in the analysis [29,30]. In turn, data driven approaches based on system variability are unbiased and considered *a posteriori* (e.g. [87]). *A priori* approaches cannot be easily transferred into *a posteriori* approaches, given that the partitioning of system variability is not transparent in *a priori* approaches.

Indicators provide a simplified and inherent measure for a complex system [88,89]. The generating process is not translation as for metaphors, but adequate representation of system information in the assessment, and accordingly indicator selection is based on sets of scientific criteria and standards [82,90–93].

Three schools of indicator selection procedures can be identified: participatory, non-reductionist and reductionist [94,95]. Participatory indicator selection reflects on democratic processes to enable stakeholder involvement and to facilitate user needs (bottom up, see [79,82]). Reductionist approaches in terms of *a priori* concepts apply theory-driven indicator development [88] or system based approaches [96], consider key areas and keystone species [91,97] or key functions (ecosystem services, [10]). Except for non-reductionist approaches (see below), indicator selection follows the principles of parsimony (few as possible) and sufficiency (covering all aspects) [36,88]. Key to the indicator selection problem in *a posteriori* structuring of ecosystem information is the non-redundant representation of system information in the assessment [88,98]. Links between indicators can exist in either way, i.e. several indicators can be linked to the same cause [39,99], and multiple causes may affect the same ecosystem component or indicator [100]. OSPAR ecological quality issues (EcoQI) and NRC ecosystem indicators represent theory driven indicator selection concepts [88] as opposed to multivariate analyses revealing key variables (redundancy analysis [101], principal components analysis [102]). Wiek and Binder [76] solved this by analyzing the indicator interaction matrix thus reweighting contributions from each indicator to the final assessment, and Samhouri et al. [103] identified links between indicators and model parameters by means of a generality index. Surrogate variables from multivariate analysis such as principal components are less informative to exact indicators due to problems with regards to time series interpretation (i.e. time series are not invariant), unclear pressure-state-response relationships (PSR) and unclear target setting [104]. One important feature of multivariate techniques is that the selected indicators can be interpreted in terms of the amount of system variability they explain. In marine assessments, only few studies considered indicator interactions and reduction, mostly addressing practical problems [102,105,106].

Non-reductionist approaches claim that the multidimensionality of sustainability assessments can hardly be condensed into one single metric and that in particular different concepts of value in economic and environmental assessments warrant the combined application of ecological and economic indicators and metrics [95]. Bossel [96] presented a theory-based approach selecting an *a priori* set of 14 indicators for each of the three sustainability dimensions (ecological, social, economic). The Bossel approach has been highly influential in socio-economic sustainability assessments in facilitating stakeholder participation and social learning [71,94].

The non-reductionist view on indicator selection leads to a tendency in sustainability assessments in moving towards an ‘indicator zoo’, where the increasing number in indicators has a reciprocally decreasing influence on decision-making [82]. The tendency to focus on individual indicators and their selection criteria generally overlooks the relevance of each indicator in the overall assessment in terms of system information contained in these indicators [89]. Consequently, the systemic dimension includes the analysis of interactions between indicators to exclude redundancy [76]. For Great Lakes assessments, the initial set of more than 800 indicators was melted down to 53 indicators [57], and for ecosystem assessments in the Baltic Sea, a special CORESET program was initiated to straighten the indicator portfolio [107].

The reduction of the number of indicators results in an increased level of abstraction for the remaining indicators in relation to the system they represent, so that “objectivity may come at the expense of usability” when specific problems are to be addressed [94].

### Indicator aggregation methods in the systemic dimension

In *ex-post* assessments, constructing a composite index is the most common way to aggregate information from the suite of selected indicators [54]. A clear policy goal is essential to develop an index, and components of the index then can be based on theory, empirical evidence or pragmatism [54,108].

Two ways of aggregating indicator information exist; i.e. additive and exclusive, and the aggregation method must be coherent with the target setting. In exclusive integration, assessments depend on either indicator *A* or indicator *B* (If *A* or *B* fail, overall evaluation is also negative). In an ecological context this either-or evaluation denies knowledge about system variability. If the sum of indicator information is considered to form an aggregate index, i.e. the integration method is additive (logical conjunction: *A* and *B*). Both methods differ in terms of their probability characteristics. In case of either conditionally independent conjunctive or disjunctive assessment elements, the overall probability of indicating a certain state *S*, i.e. *P(S)*, decreases as the number of assessment elements *X*_*i*_ increases (see [109]):

$$P(S)=\prod P(X_{i}|S)$$ (1)

This relationship from Eq 1 applies to all indicators in exclusive, but selected independent indicators in additive assessments, so that exclusive and additive assessment frameworks cannot be combined into one assessment with consistent statistical properties. Additive aggregation is the most common integration procedure to calculate indices by means of the additive utility model [54,86,110]. This has two implications, both of which refer to the indicator selection problem.

Firstly, conditional independence of variables is a prerequisite (see seminal paper from [111]), although essential criteria on independence and commensurability of indicators are often disregarded (see Discussion in [112]). Independence is defined by set theory as such that entities *A* and *B* originate from *C* (i.e. are indicators of *C*) but share no common subset:

A,B∈CandA∩B=∅ (2)

The set approach allows establishing regional and sectorial filters while assigning indicators to logically independent entities. Accordingly, any correlation between them will be spurious. Otherwise, zero conditional and partial correlations between A and B are used to indicate conditional independence (e.g. [113]).

The second aspect of additive aggregation refers to the ‘apples and oranges’ problem [110]. The optimization of the additive utility model as known from consumer theory can be described as [110,114]

$$U(X_{1},\ldots .,X_{n})=\max\;\sum_{i}w_{i}u_{i}(X_{i})$$ (3)

where *U* is the overall utility score obtained from *n* indicators, and *w*_*i*_ is the weight for the utility function *u*_*i*_ of indicator *X*_*i*_. Every indicator *X*_*i*_ has its own value function *u*_*i*_, which in order to simplify the assessment procedure can be understood as the transformation of the different measurement scales for *X*_*i*_ into an identical scale for the function *U*. This process is called normalization (with respect to *u*_*i*_) and scaling or weighting (with respect to *w*_*i*_). Differential scaling and normalization procedures account for 68% of variability in the performance of environmental indices [115]. Ebert and Welsch [116] analyzed data requirements of different aggregation methods. Their assessment criterion was the known ordering of ecosystem states which should be unambiguous in relation to different aggregation methods. As such, additive treatment can only be applied if indicators are fully comparable, i.e. have the same unit measure and scale. Incommensurable indicators mainly require multiplicative integration under certain conditions or cannot be integrated at all [116,117].

Assigning weights through expert judgment introduces a source of (un)wanted subjectivity, intuition and *a priori* knowledge to the assessment [88,108,117], and some methodologies strive at reducing the influence of expert judgment in favor of evidence based assessments [55]. If weighting and scoring is applied, the analyzed alternatives can only be evaluated in relation to each other but not in relation to a specified absolute goal or in terms of trend analysis due to the ordinal character of resulting figures ([84], 'disputable baseline problem', see [88]). Applying weights thus necessarily has the meaning of–presumed—substitution rates (trade-offs) and reflect a value judgment [77,80].

**Fig 3:**
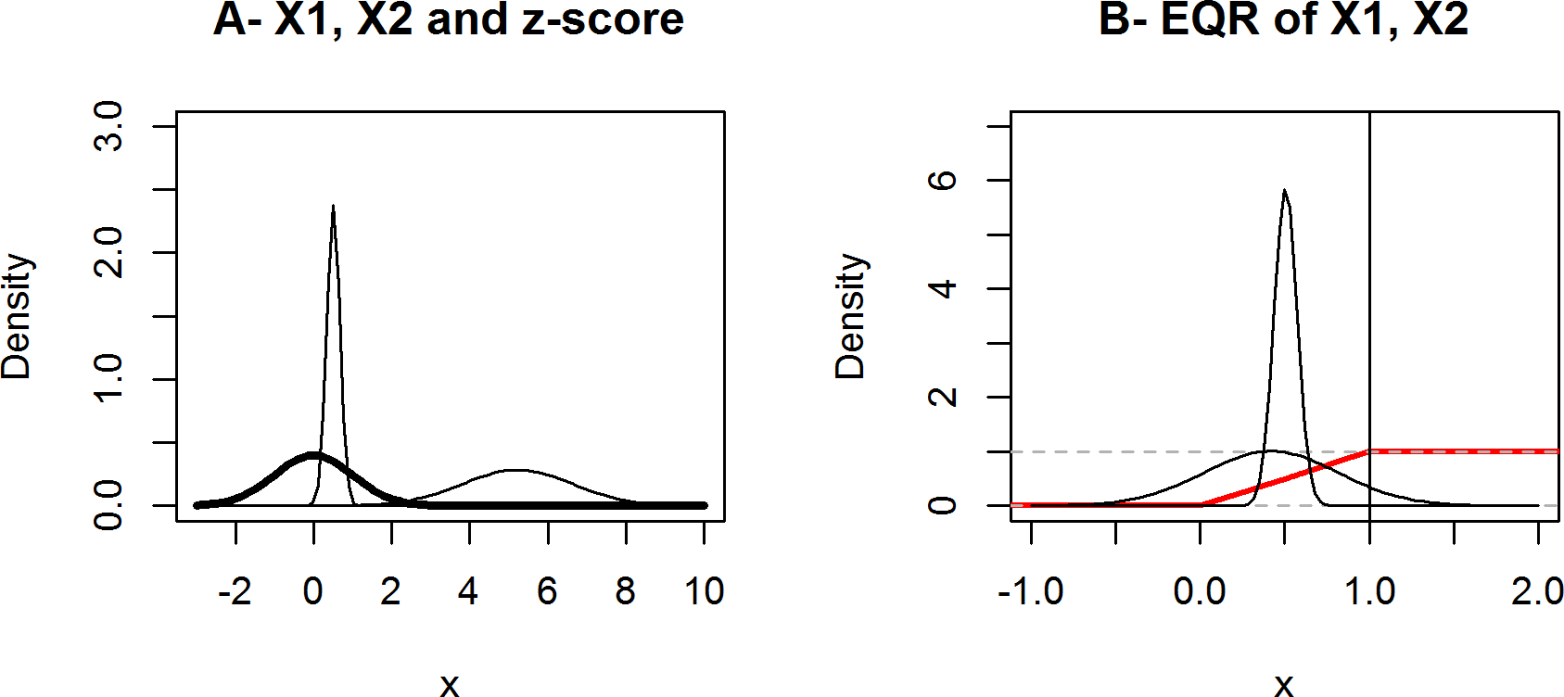
Effects of normalization procedures on data properties. (A) Two different series (X1, X2) with a 10-fold difference in value, are standardized to zero mean and unit variance (z-score, bold line), which eliminates the difference between both. (B) Normalizing to range [0,>1] (so-called Ecological Quality Ratios EQR or Contamination Ratios, resp., where 1 represents the EQR reference condition (vertical line in B)) practically changes the distribution of X1, X2 from normal to uniform [37,39]. The normalized value 0.5 for both series indicating a 50% probability of reaching the target eliminates distributional differences between both series since one indicator (narrow curve) had a real probability of 100% being below. Red line indicates uniform distribution function.

Normalization has to be considered in relation to the indicator aggregation method and whether data from the same domain (commensurable data) or different domains (incommensurable data) are analyzed. Methods such as ranking, normalizing to a range by reference value or maximum, standardizing to zero mean and unit variance (z-score) or other convenient measures [54,80,115] imply a normative value judgment [80,117]. Due to element wise normalization and pre-processing data with new properties are created (variances, order of indicators, distributions, see Fig 3), i.e. normalizing to reference value changes the scaling of data from interval (cardinal) to ratio-scale (ordinal) warranting the use of the geometric rather than the arithmetic mean in data aggregation [77,116].

In exclusive aggregation with element wise evaluations of indicators, the procedure of element wise normalization is acceptable for both commensurable and incommensurable data. In additive indicator aggregation in accordance to Eq 3, independent element wise normalization is not applicable. For indicators from different domains and to create commensurable data, a domain-generating function is required to generate values within a common domain and to normalize against a commensurable reference value (see [118]). This can be achieved by application of likelihood functions (domain = probability, see Bayesian analysis, this paper), monetary valuation and accounting techniques (domain = economic value, see [69,73]) or productivity functions [119,120]. Accordingly, comparisons can be made on cardinal scale.

## The heuristic approach for holistic assessments

**Fig 4:**
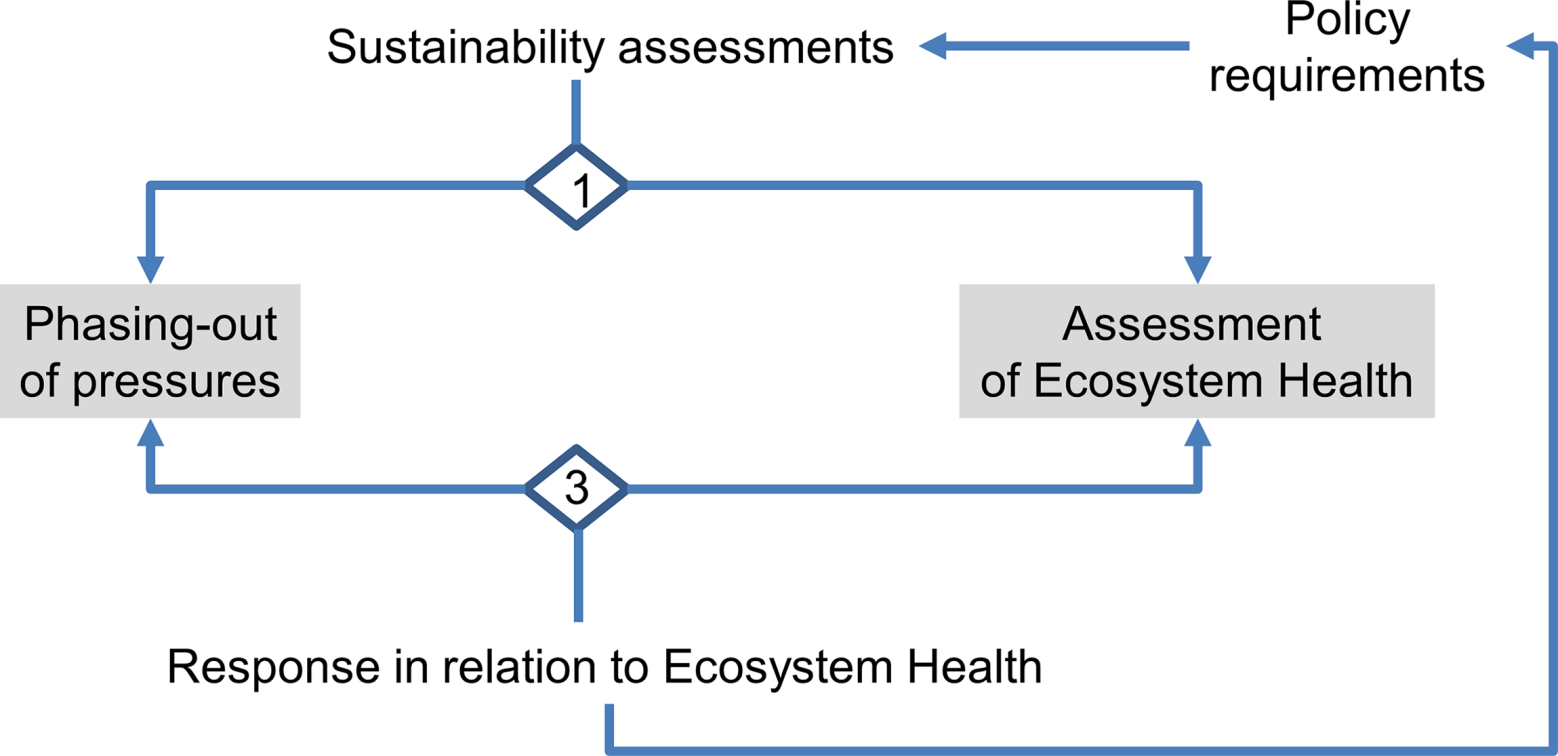
First tier level in MSFD assessments separates means related to phasing-out of pressures from the assessment of ecosystem state. Numbers refer to chapter 'European Marine Strategy Framework Directive (MFSD)'. Policy requirements need to be considered to adopt the assessment procedure, and in turn output determines the future policy needs as outlined in the canonical concept of Levin et al. [75].

The heuristic approach (hereafter HA, Fig 4) follows the outline of the canonical framework from Levin et al. [75] for ecosystem-based management including a distinct link to policy requirements as defined above, here MSFD (see S1 Text), while nesting Wiek and Binder’s [76] dimensional approach into an ex-ante/ex-post framework as a unit (hereafter EAEPNS). This combined approach establishes formal interfaces in the ex-ante/ex-post branches to confine metaphorical influence at the normative level while applying an *a posteriori* description at the systemic level by means of a formalized language, i.e. mathematical models as external reference system. HA development thus comprises five building blocks, i.e. assessment method with a corresponding normative dimension to link ex-ante modelling as phasing-out of pressures and ex-post evaluation of indicators of ecosystem health, and within each branch, choice of assessment type as systemic component, and the method of aggregation of assessment information including normalization and target setting as normative procedural component. Essentially new for HA is the development of building blocks in the ex-post branch. HA delivers two different measures, i.e. the assessment of ecosystem health in the ex-post branch and the model based outputs in the ex-ante branch.

### Detailing building blocks: The ex-ante systemic dimension

The ex-ante branch allows to analyze trade-offs as part of pressure reduction scenarios and does not need to be unified (see [121]). The multiple pressures in the phasing-out lead to the concept of causal networks as systemic dimension in the ex-ante branch ([51] with MSFD example; [89]), which requires the use of comparative tools such as risk assessments [121,122], ecosystem models [123], multi-criteria-decision-making tools [84] or economic-ecological production models [124]. These models allow for accounting for externalities as unwanted side effects, a basic element in the development of sustainability indices and sustainability assessments [125]. It implies scaling of effects in relation to reference conditions as normative component enabling straightforward target setting (e.g. maximum sustainable yield in fisheries models or gain as relation between regeneration and loss processes in risk models [120,122,126]) as opposite to un-scaled impact assessments. Scenario modeling is the means by which multi-dimensionality is introduced into the assessment.

Hence, the ex-ante branch is also the place to incorporate links to other (environmental) policies. The corresponding utility function can be rewritten as

$$U(M_{1},\ldots .,M_{n})=\max\;f(P_{1},\ldots ,P_{n}|Y_{1},\ldots ,Y_{m})$$ (4)

maximizing the utility from measures *M*_*n*_ expressed as function of pressures *P*_n_ under boundary conditions set by policy requirements *Y*_*m*_ [122]. It is evident that the solution for *U(M)* is dependent on policies *Y*, so that reaching an overall solution could compromise one part of *Y* while satisfying the other. This means that paradigmatic solutions like one-out-all-out cannot apply to the ex-ante branch (for one-out-all-out see S1 Text).

### Detailing building blocks: The ex-post systemic dimension

The HA ex-post assessment of ecosystem health is absolute with a single solution for ecosystem health following the concept of strong sustainability [22,79], considers selection of independent indicators in relation to ecosystem variability (functional-holistic) and utility models with within-domain normalization to aggregate information.

**Fig 5:**
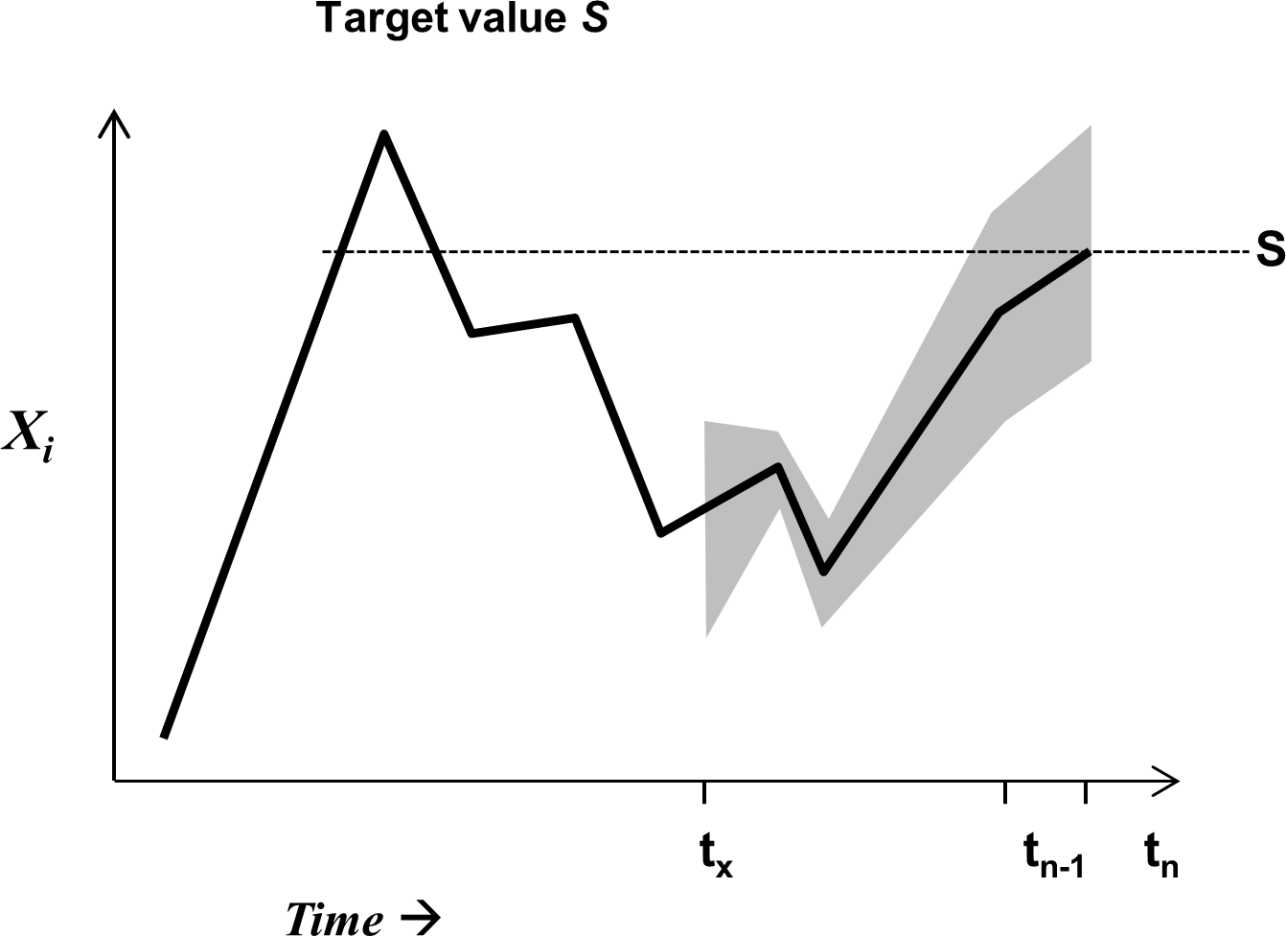
Time trajectory of indicator *X*_*i*_ representing a state indicator. *X*_*i*_ increases in the recent time period from time *t*_*n*-1_ to time *t*_*n*_ and reaches the target value *S*. The target value *S* may be pragmatically derived as some percentile of the time series [129] or from modeling [130]. Time series data have a confidence interval around the mean so that a probability distribution at any time *t* is obtained. The mean for normally distributed data indicates a 50% probability of reaching the target value S.

Indicator state at time t of indicator *X*_*i*_ (e.g. concentrations, age, weight, length) depends on the state at time *t*-1 plus new data at time *t* and thus is a time series for which change can be measured [106,127,128], i.e.

$$X_{i,t}=f(X_{i,t-1},data)$$ (5)

so that *X*_*i*,*t*_ is the mean of observations with error at time t. Each element *X*_*i*_ is assigned the probability *P*_*Xi*_ of passing (good environmental status, GES) and of 1- *P*_*Xi*_ of non-passing (non-GES) the target value. This probability is 50% when *X*_*i*,*t*_ reaches the target value (Fig 5).

In multivariate techniques to select indicators, not all variability in the data set is covered by the selected surrogate variables and they are they change with new data. Time series of indicators are invariant to new data at time t and thus the condition expressed in Eq 5 with a definite *X*_*i*,*t*-1_ is fulfilled.

As normative setting, we consider GES as function of indicators *X*_*i*_ (additive, exclusive) while applying the relationship from Eq 5.

$$U_{GES}=f(X)=f({X_{1,t-1},\ldots ,X}_{i,t-1},data)+E$$ (6)

Accordingly, *U*_*GES*_ comprises present information (data) and prior information, i.e. *X*_*i*,*t-1*_, as well as one error term *E* denoting utility that would have been obtained from unexplained ecosystem information. Evidently the associative law applies so that Eq 6 inserted into Eq 3 is a generalized description of aggregation of information across the selected set of indicators for all descriptors considered–there is no need to further elaborate on any aggregation hierarchy in information. Eq 6 can be written as likelihood function of the data, given that GES is reached, and thus likelihood serves as domain-generating function allowing us to apply Bayesian rationale. Bayesian methods reckon on all available information, present and past. The ‘data’-part of Eq 6 represents their present sampling distributions [131], i.e.

$$L(X|GES,E)=L(X_{1,data}|GES)\;*\;L(X_{2,data}|GES)\;*\;\ldots \;*\;L(X_{i,data}|GES)$$ (7)

In censored data (here: GES, non-GES), the likelihood function employs the probability distribution function instead of the density function [132]. We now ask for the overall probability of GES given the data and the available prior information *X*_*i*,*t-1*_ from Eq 6, and consider Bayes’ theorem, where the probability of *A* and *B* is the conditional probability of *A* given *B* times the probability of B, i.e. conditional dependence between indicator and GES,

P(A,B)=P(A|B)*P(B),and (8)

$$P(B|A)=\frac{P(A,B)}{P(A)}$$ (9)

Inserting Eq 8 into Eq 9 yields:

$$P(B|A)=\frac{P(A|B)*P(B)}{P(A)}=\frac{P(A|B_{l})*P(B_{l})}{\sum_{l}P(A|B_{l})*P(B_{l})}$$ (10)

The formulation of the denominator is a reformulation of Eq 8, considering the probability for *A* as sum of shared sets of *A* in conjunction with all hypotheses *B*_*l*_. This requires hypotheses *B*_*l*_ to be exclusive, which is true for the two possible states of GES and non-GES,

$$P(GES|X,E)=\frac{L(X_{data}|GES,E)*prior\;(GES)}{L(X_{data}|GES,E)*prior\;(GES)+L(X_{data}|non-GES,E)*prior\;(non-GES)}$$ (11)

**Fig 6:**
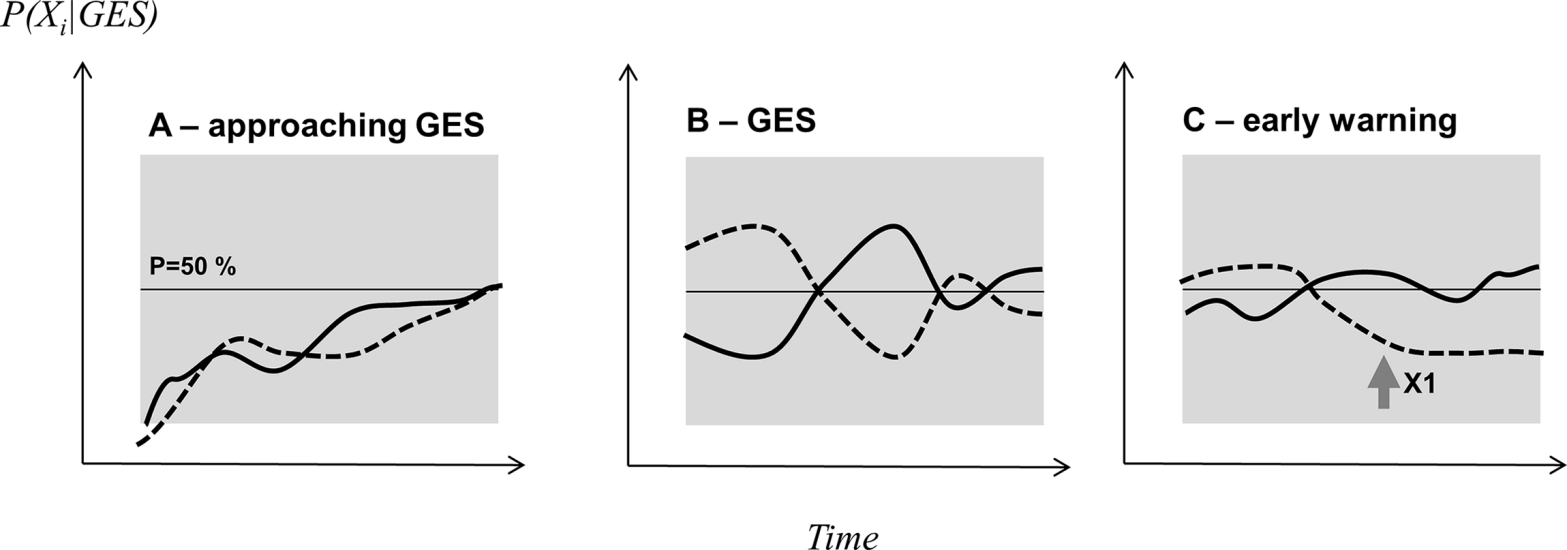
The probabilistic approach for assessing ecosystem health exemplified by means of two indicators. A 50% probability of reaching good environmental status (GES) is indicated by the horizontal lines, x-axis is time, grey area indicates state space with high probability of reaching GES. (A) Gradual approach towards GES, (B) GES state in variable environment, and (C) early warning as one indicator (X1) consistently scores low (or high).

The consideration of prior information renders Eq 11 trend-sensitive in that it 'remembers' the former state and the denominator is the within-domain normalization of Eq 3. GES is now described as probability of reaching GES given the data representing a certain amount of ecosystem variability and dynamics. The indication of probability of GES from Eq 11 provides a flexible alternative to either-or solutions as obtained from exclusive assessment methodologies, in particular in a stage when the system is improving but has not reached GES (see Fig 6A).

The increase of objectivity by reducing the number of indicators to obtain a probability measure of GES does not necessarily lead to a reduction of user-friendliness due to an increased level of abstraction which has been argued in the literature [94,133]. Eq 7 shows that GES can always be traced back to the individual indicators and their likelihoods and the corresponding indicator groups and thus the degree of variability explained by these selected indicators in the indicator space (S1 Appendix).

## HA in relation to existing assessment methodologies and policy frameworks

### European Marine Strategy Framework Directive (MSFD)

The goal of MSFD is to attain ecosystem health by means of good environmental status (GES) in EU marine waters by 2020. At European Commission level, this is understood as a yet undefined combination of indicators into one measure of GES [52,59,104]. Specifications of the MSFD as detailed in the respective legal documents (S1 Text) indicate that a deconstructing structural assessments type is pursued based on 11 descriptors of ecosystem integrity (S1 Table), with two specific forms of policy measures, i.e. one related to assessing ecosystem and the other related to a remediation of human pressures. Nineteen assessment methods have been proposed to date for MSFD assessments (S2 Table), based on 56 generic indicators which have been specified to 557 indicators at species and habitat level [134]. Mainly mixed concepts are advocated, with exclusive aggregation for pressure and additive aggregation for state indicators but without guidance how to achieve the overall assessment. The method for the exclusive assessment is one-out-all-out (OOAO, see S1 Text).

**Fig 7:**
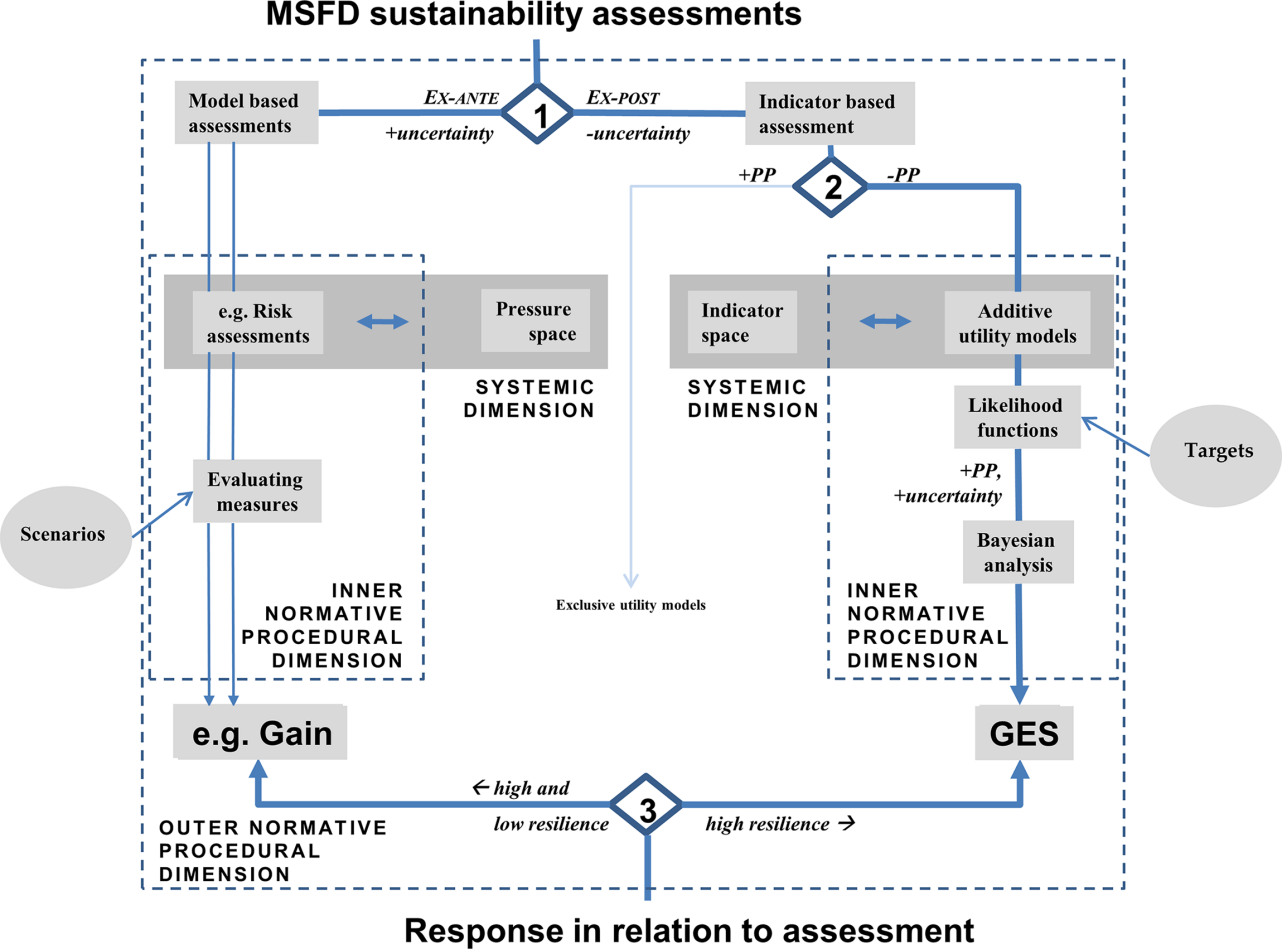
Detailed assessment tree following the EAEPNS structure for HA sustainability assessments to indicate good-environmental-status (GES) under the Marine Strategy Framework Directive (MSFD). Steps 1–3 explained in text, steps 1 and 3 refer also to Fig 4. Circles indicate external normative inputs. OOAO–one-out-all-out, + or -PP–preserving or abandoning the precautionary principle.

HA resembles the basic structure of the MSFD, i.e. the assessment of GES in the ex-post branch and the model based outputs in the ex-ante branch (Fig 7 step 1). The difference between ex-ante and ex-post assessments indicates system resilience and is the key to adaptive management. For each ecosystem component, a respective succession-time trajectory has to be anticipated [122], so that the ex-ante assessment will apply to features with both high (rapid recovery) and low resilience (slow recovery), whereas the ex-post assessment will best apply to features that are very responsive to changes of pressures (see [41]). The potential of combining both approaches for MSFD purposes was recognized, when stating that risk based and indicator based assessments could be applied to pressure and state descriptors differentially [51,104].

The systemic dimension identifies the relevant pressures and indicators for each of the branches, either in terms of causal networks of pressure relationships (pressure space) or in terms of the analysis indicator space to select indicators based on the criteria of independence and ecosystem variability they represent (*a posteriori* selection). They can be linked to a hierarchical concept, but this is not essential for the assessment. The selected indicators receive no particular weighting.

At step 2 (Fig 7), the treatment of uncertainty determines whether exclusive or additive assessment methods are chosen. Exclusive assessments (e.g. OOAO) are non-reductionist precautionary tools and thus try to incorporate indicator information as much as possible with high costs of implementation [78]. The probability of rejecting a true GES state as incorrect is high (Eq 1, type II error). This does not apply to additive models with a subset of conditionally independent indicators. In additive models, the precautionary principle is re-introduced by means of precautionary target setting in the likelihood function, i.e. targets can be developed to be either disturbance friendly or environmentally friendly, i.e. precautionary (see [135]).

The Bayesian model (Eq 11) provides a probability value of reaching GES, now integrating uncertainty and PP. The inclusion of prior information in the Bayesian model enables easy tracking of incremental changes in selected indicator ensembles ranging from 0% to 100% (Fig 6A). Accounting for subtle changes in ecosystem state and trend information is one of the key challenges in ecosystem based management [59,136].

The same GES probability (Fig 6B) can be obtained by different combinations of likelihoods from the individual indicators (S1 Appendix). Thus, indicators are allowed to vary within a given range to account for stochastic variability and short-term perturbations in ecosystem properties. This is consistent with methods that account for an allowable range of indicator values [86,137] and the state space approach to evaluate ecosystem health [41]. In Eq 7, consistently under- or over-scoring in likelihoods over a certain period of specific indicators would indicate persistent changes in certain ecosystem components while not in others. This would allow to install an early warning system ([138], see Fig 6C). Both, the capability to deal with unequal indicator probabilities and the buffer towards ecosystem variability equip the Bayesian model with two realistic features to assess ecosystem state.

### OSPAR, HELCOM and Water Framework Directive (WFD): A worked example

**Fig 8:**
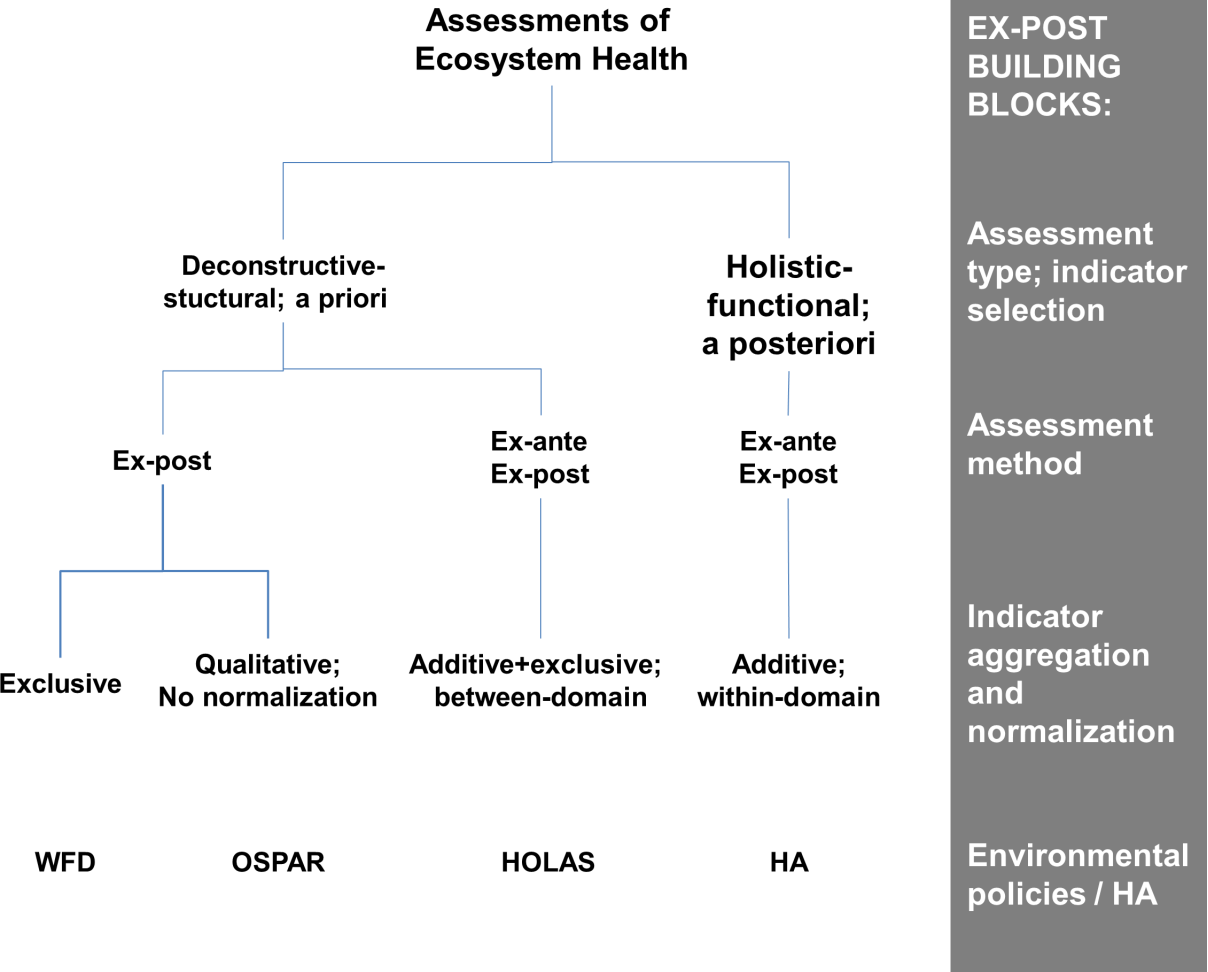
Decision tree of existing methodologies for assessing ecosystem health and the heuristic approach (HA). Screening is undertaken with respect to building blocks of the ex-post heuristic approach. Assessment methodologies are described in S3 Table. HOLAS–Holistic Assessment for the Baltic Sea, WFD–Water Framework Directive assessments, OSPAR–OSPAR ecosystem assessments

Assessments undertaken by regional conventions OSPAR and HELCOM and the European Water Framework Directive screened with regards to HA building blocks of assessment type, assessment method and indicator aggregation and normalization indicate significant differences in relation to HA (Fig 8, S3 Table). OSPAR, HELCOM and WFD apply the deconstructive-structural assessment type with *a priori* selection of indicators, i.e. the amount of ecosystem information contained in these assessments is unknown. Only one methodology comprises an ex-ante/ex-post structure, i.e. the HELCOM ecosystem assessment HOLAS (see Fig 1). OSPAR and the WFD apply indicator-based assessments, although OSPAR assessments include trends of ecosystem states and pressures as parameters in the ex-post assessment, based on expert judgment rather than detailed trend modelling.

With regards to aggregation methods, WFD applies exclusive aggregation (one-out-all-out). HOLAS applies a combination of additive and exclusive indicator aggregation without considering the associated statistical problems, and weighting by means of expert judgment. HOLAS and WFD both undertake between-domain normalization with subsequent changes of the properties of the input variables (see Fig 3). OSPAR assessments are different in that they apply qualitative, i.e. narrative aggregation without any normalization of the time series data, so that their statistical properties are maintained [55]. A first holistic assessment approach, i.e. the ‘Utrecht Workshop’ methodology, applying exclusive aggregation methods is considered preliminary [55,62].

A worked example for HA based on OSPAR assessments reveals (S1 Appendix) that HA cannot be applied to existing assessment frameworks without difficulties. OSPAR assessments are based on Ecological Quality objectives (EcoQO) which provide a link between human activities and impacts on biodiversity, but also specify the desired state of an ecological component or mechanism [55,139]. This would allow to consider EcoQOs within a PSR framework, but since PSR relationships are not always clear [139], also as part of an indicator-based *ex-post* assessment (see S3 Table). In line with the latter, the southern North Sea with the adjacent Wadden Sea area is one of the few regions within the OSPAR area to have sufficient data coverage published in OSPAR and Wadden Sea status reports. Since 6 out of 11 OSPAR EcoQOs directly refer to Wadden Sea quality objectives, OSPAR and Wadden Sea status reports are acquired as data sources to create an indicator space for the southern North Sea. Multivariate analysis based on 36 deliberately selected variables explains 57% of variance by the first two principal components (PCA1, PCA2), with 44% of variance assigned to PCA1, indicating a high degree of correlation between the variables as a matter of their joint responses to maritime environmental policies and/or pressures; they are logically dependent. Variables dependent on river loads were assigned to PCA2, accounting for 13% of variance. In redundancy analysis, 42% of variance can be explained from 2 variables, i.e. large fish indicator (LFI) representative of PCA1 and ppDDE concentrations in bird eggs related to PCA2. Since ppDDE is an ecosystem parameter but not an element of the respective EcoQO with a corresponding target value, a tentative target value was calculated for the purpose of this example for ppDDE based on the DDT target given that ppDDE is a breakdown product of DDT. Depending on the prior, likelihood of reaching target values for the years 2007 and 2008 was <0.01% (Table 2) with two reservations, i.e. the ppDDE value was taken from the most contaminated site, the island of Trischen, whereas other sites were already below the tentative target value in both years, and secondly, that 58% of ecosystem variance were not explained. Simulations with increasing trend (simulations II and III) and decreasing trend (simulation IV) indicate conservative behavior of the GES index due to the effect of the prior. The respective OSPAR 2010 assessment concluded, that EcoQOs for fish populations, the large fish indicator (LFI), and contaminants in bird eggs eutrophication were not met, and partly met for seals and for oiled seabirds [38]. The following EcoQOs were not included in the analysis: Plastic particles in bird stomachs (short time series), imposex in dogwhelks (not operational), harbor porpoise by-catch (no local data).

**Table 2:** Worked example for OSPAR EcoQOs for the southern North Sea based on two EcoQO elements, LFI and ppDDE (Trischen). Explained variance in simulations not indicated. Data and calculations in S1 Appendix.

Model specification	Likelihood GES 2007	Likelihood GES 2008	Explained ecosystem variance
Real values 2007 and 2008; prior 2007 = 0.01	<0.01%	<0.01%	42%
Simulation I: ppDDE on target, LFI real values	<0.01%	<0.01%	NA
Simulation II: both first year on target in 2007, 2007 = 2008, prior 2007 = 0.45	45.0%	45.1%	NA
Simulation III: Both first year at target +10% in 2007, 2007 = 2008, prior2007 = 0.45	59.7%	72.9%	NA
Simulation IV: Both first year at target -10% in 2007, 2007 = 2008, prior 2007 = 0.55	40.9%	28.2%	NA

## Discussion

### Metaphors in the Heuristic Approach

The question in evaluating metaphors is whether the use of metaphors is misleading in scientific terms. Hierarchical metaphors in deconstructive-structural assessments promote assessments that do not indicate the amount of ecosystem information explained but rely on *a priori* knowledge, which is expressed in terms of confidence in assessments [62,67], of certainty in relation to values [112], and of importance of indicators [10,92,105]. Further normative components include *a priori* indicator selection, aggregation of partially redundant indicators, between-domain normalization of indicators and associated weighting and scoring procedures. HA instead builds a generic framework based on five building blocks where metaphorical influences are minimized: an ex-ante/ex-post framework as outer normative-procedural dimension, and inner normative-procedural and systemic dimensions for each of the branches (EAEPNS). The systemic dimensions in each of the branches provide a built-in check of system variability. Thus the basic difference between deconstructive-structural assessments and HA may be seen in the treatment of explained system information, which in HA is the variance model embedded in the additive utility function (Eq 6) or uncertainty in risk models.

HA employs two metaphors. The metaphor in the functional-holistic assessment is the ecosystem as entity. This metaphor is inconsequential, since the additive utility model is open and the only basis to select indicators in the ex-post branch is their non-redundant information contents retrieved out of observable system dynamics: If a system consisted of two or more independent subsystems, this would be revealed in the *a posteriori* analysis and a respective change in the indicator portfolio (see worked example). In the same way HA is open to extended concepts integrating health and environmental impact assessments (i.e. HIA/EIA, [140,141]), leading to more indicators in the ex-post branch and respective pressure chains in the ex-ante branch. The second metaphor in ecosystem health implies that there is a certain properly measurable ecosystem state, for which a reference level exists for target setting. This is challenged by the view, that ecosystems are dynamic self-organizing adaptive systems [48,63]. Intrinsic ecosystem dynamics can lead to self-organized criticality, generating new structures in the ecosystem so that envisaged gradual changes in ecosystem properties (Fig 6A) are replaced by abrupt changes, i.e. ‘tipping points’ [63]. Management of systems with changing stability conditions needs adaptive target setting [64], which can be accounted for in the target setting procedure, but not necessarily is a matter of changing the assessment structure (see Fig 6C).

### HA as methodology

The most palpable element of HA is the building block containing the ex-ante/ex-post framework with the associated inner normative procedural and systemic dimensions of each branch (EAEPNS framework). It provides a means for translation of a policy aim into a scientific tool, capable of adaptive management and the analysis of policy scenarios. Mee et al. [78] present a similar two pillar approach for comprehensive management of human activities that reflects the ex-ante/ex-post part of EAEPNS, addressing in particular the different behavior of slow and fast variables. However, in most publications on ecosystem health assessments, the distinction between parallel ex-ante and ex-post assessments is not made in favor of linear schemes of increasing complexity from indicator based assessment to model based assessments [35,47]. The Ocean Health Index [10] can be seen in an intermediate position, combining modeling and indicator assessments in one index. It models ecosystem state as mean of present state and future prospects, including a trend factor and the difference between pressures and resilience and thus in part resembles the structure in risk models (but difference approach [10] vs ratio approach [120,122,126]).

The separation into two branches is essential to provide an ex-post ecosystem index under the concept of strong sustainability, whereas ex-ante assessments in particular tend to provide indices under weak sustainability (in [77]). As explained earlier, the choice between weak and strong sustainability determines the choice of assessments in the outer normative-procedural dimension [69]. The inclusion of resilience for 'slow variables’ is the probable difference to actively adaptive management schemes as outlined by Linkov et al. [84],where modeling, implementation of measures and monitoring are treated as subsequent steps which requires an instant response of the managed system, but would be insufficient to treat 'slow variables'.

**Fig 9:**
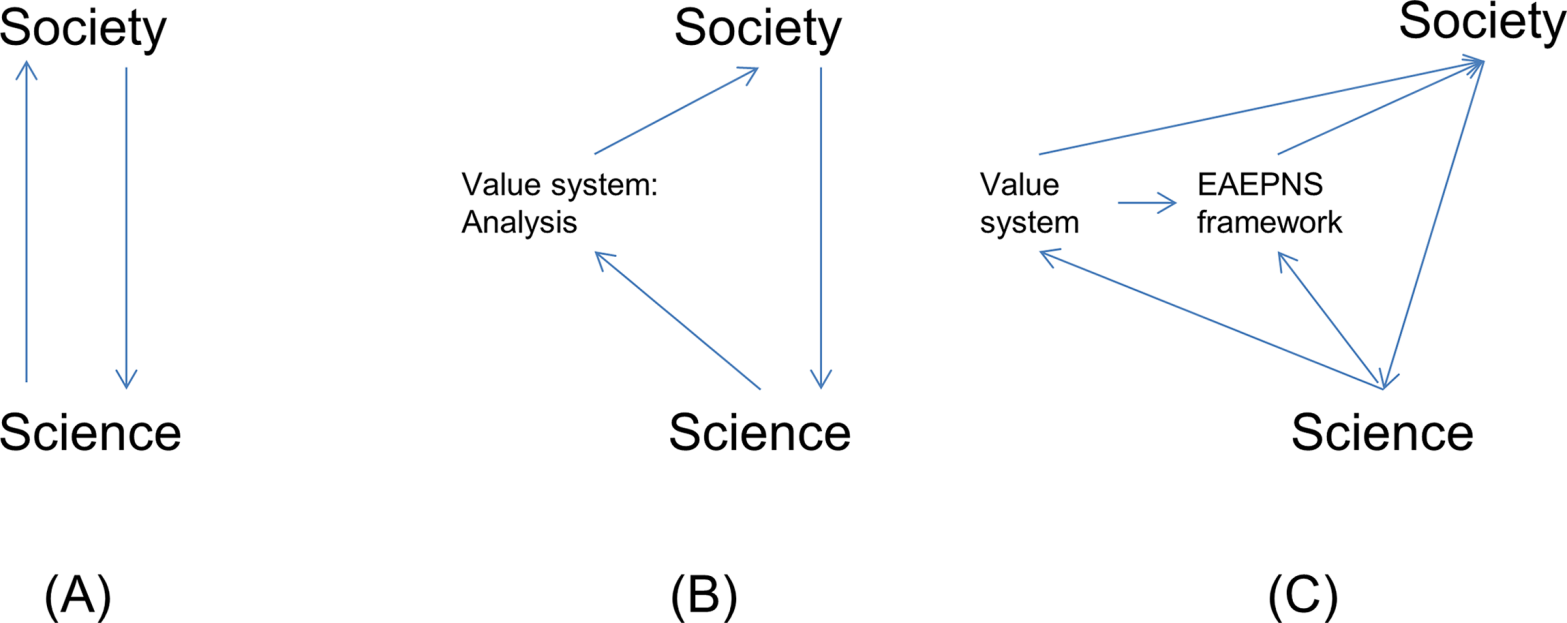
Modification of Larson's 'feedback metaphor'-model under the heuristic approach. (A) Environmental metaphor understanding is generated through multiple feedbacks between society and science [5]. (B) Obligation for science to assess public values associated with a metaphor to avoid misleading metaphors [5,144]. (C) The EAEPNS framework as interface for assessments between science and society.

In both branches valued metaphoric expressions were transformed and replaced by systemic, algebraic contents. In HA, the normative element in setting up a model is further balanced by the need to express its uncertainty and statistical properties. In the worked example, the redundancy analysis showed to what degree system dynamics were represented in the model. The replacement of value-laden metaphoric contents with algebraic expressions follows the line of argument from Lackey [24] stating that the most straightforward alternative to metaphors is the simple and clear description of what is proposed. Similarly, Pickett et al. [142] for the metaphor ‘ecosystem’, Hurlbert [143] for the metaphor ‘keystone species’, and Mikkelson [16] exploring the application of machine metaphors in community ecology, suggest the use of formalistically defined functional or algebraic expressions to avoid ambiguous metaphor contents. Referring to Schön's 3-stage model for applying generative metaphors in policy making [18], this formalistic 'grounding' refers to stage 2, making metaphors operational within a policy framework. However, in the literature on metaphor applications much stronger emphasis was laid on the stage 1 process of identifying a problem and phrasing the appropriate metaphor. Larson’s [5] ‘feedback metaphor’-model describes the interference between society and science in the evolution of an interaction metaphor (Fig 9A). Public values behind metaphors need to be assessed in order to avoid metaphors to become misleading (Fig 9B), hence to apply 'appropriate language' ([19], p. 229) or 'language planning' [17].

In interaction metaphors both the principal subject and the subsidiary subject consist of ‘systems of things’ rather than of plain ‘things’, and the user has to apply a “system of implications as a means for selecting, emphasizing, and organizing relations in the (two) different fields” [14]. The EAEPNS framework can be understood as tool to facilitate this process. Implementing EAEPNS as further ingredient into Larson’s [5] ‘feedback metaphor’-model creates a by-pass for assessments independent from societal prerogatives but with a link to value system analysis (Fig 9C). Likewise, Sarewitz [4] also advocates a clear separation of scientific contents from societal value system, and that scientific progress in solving environmental problems occurs only after adjudicating on the value system.

Bayesian methods as applied in the ex-post assessment are the only tools to test multiple hypotheses simultaneously (e.g. GES, non-GES, [109]), and are a mathematically consistent way of incorporating information into decision making processes [131,145]. The likelihood function without weighting of components reflects Black’s statement [19] that in terms of ‘grammar’ (algebra) there is no consistent method to distinguish between ‘ontologically essential units’ (high weight) and 'non-essential parts of language' (low weight) without introducing an extra-grammatical concept (applying weights). The ex-post assessment delivers one value of ecosystem health, normalized in terms of overall probability and related to explained ecosystem variability. The link to ecosystem variability defines the functional-holistic character of HA, since ecosystem functioning is evident in the variability of its components. While accounting for multi-dimensionality in the ex-ante branch, HA thus avoids problems of condensing information of the entire social-ecological system into one sole index [77,86,95].

### Feasibility of HA

A transition from WFD, HOLAS, and OSPAR and other methodologies to HA though difficult (see worked example) can be considered by adopting EAEPNS building blocks to existing concepts. Firstly, a distinct separation in ex-ante and ex-post branches could be undertaken. This would partly relieve the pressure-state-response (PSR) paradigm in environmental policies from the burden of providing explicit knowledge on pressure-state relationships where these are treated as indicators. PSR chains in practice are hard to interpret due to combined effects of anthropogenic pressure and natural variability including resilience and the multiple links between state indicators and pressures. Therefore the pressure side is more effectively treated in modeling reduction scenarios of the ex-ante branch (e.g. [122]). Secondly, a ‘*re-posteriorization’* could be considered to shift from deconstructive-structural to functional-holistic assessments, which means the analysis of available indicator space and indicator interactions in relation to ecosystem variability. Thirdly, normalization could be undertaken by means of Bayes' theorem and likelihood as domain-generating function to obtain a probability value without arbitrary scaling and weighting.

Establishing an ex-ante/ex-post framework in favor of the concept of strong sustainability would be achievable at low costs for society. The second process, i.e. *re-posteriorization*, would require considerably more effort to monitor ecosystem variability with sufficient coverage in the future, which has been identified as a significant impediment for ex-post based assessments of ecosystem health and associated socio-economic parameters [57,60,78,146,147]. The worked example shows that sufficient data coverage was available only where OSPAR and Wadden Sea assessments overlapped. Accordingly, Heslenfeld and Enserink [139] report on the hesitant commitment of North Sea countries to contribute effort to monitoring the even smaller list of OSPAR EcoQOs, and data demanding approaches such as the Multiple Marine Ecological Disturbance program [148] proved to be not successful in the long-term. Whereas from a societal viewpoint it could appear questionable to further expand effort for monitoring of the entire ecosystem for functional-holistic assessments, from a scientific viewpoint this means to abandon the concept of functional-holistic assessments where the entire ecosystem cannot be analyzed. Thus opposite to expanding monitoring effort as first solution, the second way ahead could be applying HA with an emphasis on the ex-ante branch (see [11]), applied against a background of unknown total ecosystem variability but still with a necessary understanding of change in the ecosystem towards ecosystem health. The ex-post component would then provide evidence from well-studied subsystems, e.g. OSPAR EcoQOs, applying *a posteriori* indicator selection and avoidance of redundancy, additive aggregation and Bayesian normalization, while modeling would reflect pressure space in the ex-ante branch [89]. This resembles the 'pressures only'-option in solving problems in MSFD assessments [59]. Appropriate language planning would be required to put a focus on for instance EcoQOs instead of ecosystem health, where the normative decision would be to accept information from well-studied subsystems as being representative of the entire ecosystem. Besides existing time series like OSPAR EcoQOs [38], fisheries information could provide reliable long-term data base for such assessments in marine ecosystems, e.g. IndiSeas project [93,101,149]. A third solution could be to further down weight science driven aspects in favor of governance driven approaches and build upon participatory processes and stakeholder involvement as described for indicator selection (bottom-up, integrated participatory approach [36]). Increased stakeholder participation will promote regional approaches [36] and could positively influence achieving conservation targets [150]. This was shown for the assessment of the Bay of Fundy ecosystem [151]. Selection and trend based assessment and management of key indicators was consensually agreed upon by all stakeholders [151], comparable to procedures in OSPAR and HOLAS assessments. Accordingly, principles for building resilience in ecosystems as part of sustainability strategy as outlined by the Resilience Alliance Young Scholars network [152] put a strong emphasis on governance. The principles comprise to maintain diversity and redundancy (P1), manage connectivity (P2), manage slow variables and feedbacks (P3), foster complex-adaptive-system thinking (P4), encourage learning (P5), broaden participation (P6) and promote polycentric governance systems (P7). P1-P3 could be ideally treated within the ex-ante/ex-post framework of HA, with P1 being a typical ex-post and P2 an ex-ante assessment, whereas P3 could be inferred from the differences between both branches (see Fig 7). Principles P4-P7 refer to the governance system of the social-ecological system and thus are part of the value system of ecosystem health according to Rapport [70].

## Conclusion

In the face of high risks for society and environment, society urgently seeks decision support from scientific tools that can deal with high degrees of uncertainty and data limitations. Several authors have thus claimed to develop a ‘post-modern’ science [23,153]. However, the reconstructive metaphor analysis and the subsequently developed heuristic approach show, that tools are at hand to cope with assessment problems within an ex-post/ex-ante framework to integrate scientific knowledge into decision making. In particular the need to have assessments under the primacy of strong sustainability asks for carrying out ex-post assessments in relation to explained ecosystem variability. Regionalized and governance driven assessment and decision making processes can also be linked to the HA framework, accompanied by ex-post assessments in well studied sub-systems which may play a sentinel role revealing how resilient the ecosystem is in responding to changes in human pressures. The separation between ex-ante modeling and analysis of complexity in the entire indicator space and ecosystem variability resembles the bifurcation foreseen by Hilborn [154] for fisheries management, stating that future management decisions will be based on simple rules and models rather than on complex models which in turn have their value in checking the robustness of the simpler assumptions.

## Supporting Information

S1 Appendix (2 files) Worked examples of an assessment of Good Environmental Status (GES) in the ex-post branch in relation to (a) a theoretical case study focusing on one-out-all-out (OOAO) and the heuristic approach (HA) and (b) an application of HA to OSPAR data and EcoQOs for the Southern North Sea
(ZIP)

S1 Table MSFD qualitative descriptors for determining good environmental status. As indicated in European Commission Directive 2008/56/EC Annex I.
(DOCX)

S2 Table Approaches proposed for the MSFD for aggregation of different metrics, indicators or criteria to assess good environmental status, including the advantages and disadvantages of each approach, covering modeling and indicator based methods. (DOCX)

S3 Table Comparative screening procedure for selected maritime environmental ecosystem health assessment methodologies. Screening is undertaken with respect to three procedural steps essential to the heuristic approach.
(DOCX)

S1 Text MFSD assessment framework. (DOCX)
