# Supplementary material for: From Metaphors to Formalism: A Heuristic Approach to Holistic Assessments of Ecosystem Health
Source: PLoS One. 2016 Aug 10;11(8):e0159481. doi: 10.1371/journal.pone.0159481 (PMC4980027; doi:10.1371/journal.pone.0159481)

S1 Appendix

Supporting information to

From metaphors to formalism: A heuristic approach to holistic assessments of ecosystem health

Heino O. Fock, Gerd Kraus

[S1 Appendix: Worked examples of an exemplified assessment of Good Environmental Status (GES) in the ex-post branch in relation to (a) a theoretical case study focusing on one-out-all-out (OOAO) and the heuristic approach (HA) and (b) an application of HA to OSPAR data and EcoQOs for the Southern North Sea 2](#_Toc457290007)

[OOAO and HA 2](#_Toc457290008)

[OSPAR EcoQOs for the southern North Sea 3](#_Toc457290009)

[References 4](#_Toc457290010)

## S1 Appendix: Worked examples of an exemplified assessment of Good Environmental Status (GES) in the ex-post branch in relation to (a) a theoretical case study focusing on one-out-all-out (OOAO) and the heuristic approach (HA) and (b) an application of HA to OSPAR data and EcoQOs for the Southern North Sea

The first worked example considers the differences between exclusive (OOAO) and additive aggregation according to HA. The second example applies HA to a deliberate selection of OSPAR ecosystem trend data and EcoQO for the southern North Sea.

### OOAO and HA

Consider 10 indicators belonging to one criterion, each with a probability of P_Xi_=0.5 of reaching GES. This means that the mean (median) of indicator X_i_ reaches the desired bound of the target value distribution (targets also have a distribution).

(1) With 10 elements considered, the overall probability of indicating GES at *A_1_* by means of OOAO is <0.001, i.e. in more than 99.9 % of all cases non-GES will be indicated, although all elements have reached the target value. This means that in a 56+X framework the probability of reaching overall GES will be infinitely small except for in cases where all probabilities of reaching GES are almost 100%.

(2) Due to redundancy analysis, the number of effective indicators is reduced to 3 and 7 correlated indicators are excluded (not a prerequisite for OOAO). With three indicators, the accordingly calculated probability of indicating GES correctly by means of OOAO is 0.125, and in 87.5 % of all cases the analysis fails to indicate GES, indicating a high type II error.

(3) In the Bayesian model, assuming that the same conditions also prevailed for the previous years to define the prior, the probability of GES is:

$\boldsymbol{P}\left( \boldsymbol{GES} | \boldsymbol{X}_{\boldsymbol{i}} \right)\boldsymbol{=}\frac{\boldsymbol{L}\left( \boldsymbol{X}_{\boldsymbol{i}}\boldsymbol{|}\boldsymbol{GES}\boldsymbol{,E} \right)\boldsymbol{*}\boldsymbol{prior} \left( \boldsymbol{GES} \right)}{\boldsymbol{L}\left( \boldsymbol{X}_{\boldsymbol{i}}\boldsymbol{|}\boldsymbol{GES}\boldsymbol{,}\boldsymbol{E} \right)\boldsymbol{*}\boldsymbol{prior} \left( \boldsymbol{GES} \right)\mathbf{+}\boldsymbol{L}\left( \boldsymbol{X}_{\boldsymbol{i}}\boldsymbol{|}\boldsymbol{non}\boldsymbol{-}\boldsymbol{GES}\boldsymbol{,}\boldsymbol{E} \right)\boldsymbol{*}\boldsymbol{prior} \left( \boldsymbol{non}\boldsymbol{-}\boldsymbol{GES} \right)}\boldsymbol{=}\frac{\boldsymbol{0.125*0.5}}{\boldsymbol{0.125*0.5+0.125*0.5}}$

This result 0.5 would also be expected from inspection of the indicator data themselves, and serve as prior for the next assessment.

Consider variability in the indicators, the probability according to eq. 11 is preserved as long as balanced under- and overshooting in individual indicator probabilities occurs. For instance, with likelihood functions of 0.4*0.6*0.5 and for non-GES of 0.6*0.4*0.5, the overall probability still is 50 % applying the priors mentioned before, whereas OOAO would not indicate GES in either case given that there is one non-GES in the row. Thus, the Bayesian approach allows to accept variability in ecosystem properties. As a rule of thumb it may be assumed, that the target value should fall well within the indicator’s CIs, so that no zero probabilities are obtained.

### OSPAR EcoQOs for the southern North Sea

OSPAR assessments are regionalized, either with regards to availability of time series data or with regards to regional differences in the performance of indicators. The EcoQO for eutrophication is assessed for small sub-regions within regional seas, reflecting regional inputs and turn-over processes. As such, the southern North Sea is considered a 'problem area' for eutrophication, based on the relatively high riverine inputs of nutrients to the area including the Wadden Sea. EcoQOs for contaminants in bird eggs, seals (partly), and oiled seabirds found ashore also refer to the southern North Sea and the Wadden Sea. Selecting time series of EcoQO indicators and ecosystem parameters from the southern North Sea and the Wadden Sea was therefore chosen as approach to exemply an ex-post HA assessment and the analysis of indicator space.

36 variables were chosen comprising EcoQOs and very different ecosystem components representative of the southern North Sea and Wadden Sea (habitat or population trends) to exemplify ecosystem dynamics and thus indicator space. Not all EcoQOs apply to the selected area, nor are all EcoQOs operational (e.g. imposex), and selected EcoQOs only cover a small suite of sampled locations and parameters in this area (Table S1App.1). For instance, contaminants in bird eggs are sampled in 12 locations with available trend data, however, only data from the island of Trischen were selected. For a full list of EcoQOs see supplementary material S5. Data references for fish and PHI40 [1], OSPAR and Wadden Sea data [2–6].

Multivariate analysis (Table S1App.2) revealed strong correlations between variables along two axes covering 57 % of total variance (Fig. S1App.1). For the first axis this can be explained by correlated policies in all fields of OSPAR maritime protection, whereas the second axis indicates an independent influence as evidenced by river loads, i.e. influence of meteoclimatic conditions. Concentrations of ppDDE in eggs of Common tern (CT_ppDDE) at the island of Trischen appear correlated with river loads. RDA with LFI and Phosphate river loads as explanatory variables explains 48 % of indicator space, but river loads have no EcoQO. Therefore the DDT derivate ppDDE was applied, with LFI and ppDDE explaining 42 % of ensemble variance.

Both indicators are far from their respective target values, although with regards to organochlorine contaminants Trischen is much higher than than the non-estuarine habitats in the Wadden Sea.

The resulting likelihood of reaching GES resp. OSPAR target value is <0.001 %. Two additional runs to reveal trend sensitivity are explained in the text.

## References

1. Fock HO, Kloppmann M, Probst WN. An early footprint of fisheries: changes for a demersal fish assemblage in the German Bight from 1902-1932 to 1991-2009. J Sea Res. 2014;85: 325–335. doi:dx.doi.org/10.1016/j.seares.2013.06.004

2. Greenstreet SPR, Rogers SI, Rice JC, Piet GJ, Guirey EJ, Fraser HM, et al. A reassessment of trends in the North Sea Large Fish Indicator and a re-evaluation of earlier conclusions. ICES J Mar Sci. 2012;69: 343–345.

3. OSPAR Commission. Evaluation of the OSPAR system of Ecological Quality Objectives for the North Sea (update 2010). London; 2009.

4. Wolff WJ, Bakker JP, Laursen K, Reise K. The Wadden Sea Quality Status Report - Synthesis Report 2010. Wilhelmshaven; 2010.

5. Graaf S van der, Vlas J de, Herlyn M, Voss J, Heyer K, Drent J. Macrozoobenthos, Thematic Report No. 10. Wilhelmshaven; 2009.

6. Becker PH, Dittmann T. Contaminants in Bird Eggs, Thematic Report 5.2. Wilhelmshaven; 2009.

#### Table S1App.1 : List of selected parameters and annotations

B1-B3 – three species selected to represent negative and positive trends; CT_contaminants – only data from island of Trischen used; LFI – applies to entire North Sea, highly correlated with PHI40, which is calculated as local index; VT – saltmarsh area in Schleswig-Holstein (Hamburger Hallig); f – abundance indices of typical fish species for the southern North Sea as proxy for respective stock sizes

| Acronym | Ecosystem parameter | Description |
| --- | --- | --- |
|  |  |  |
| B1_CT | Common tern | Population trend annual change |
| B2_LBBG | Lesser black backed seagull | Population trend annual change |
| B3_SRP | Snowy plover | Population trend annual change |
| CT_HCB | HCB in eggs Common tern | EcoQO |
| CT_Hg | Hg in eggs Common tern | EcoQO |
| CT_PCB | PCB in eggs Common tern | EcoQO |
| CT_ppDDE | ppDDE in eggs Common tern | Ecosystem parameter |
| GM_oilrates | Rates oiled guillemots | EcoQO |
| LFI | Large fish indicator | EcoQO |
| LoadN_Elbe | Nitrogen load , Elbe | Ecosystem parameter |
| LoadN_Rhine | Nitrogen load , Rhine | Ecosystem parameter |
| LoadP_Elbe | Phosphate load , Elbe | Ecosystem parameter |
| LoadP_Rhine | Phosphate load , Rhine | Ecosystem parameter |
| SG% | Sea grass coverage in Wadden Sea Schleswig-Holstein | Habitat trend |
| Seals_NLD | Trend Grey seals , Dutch area | EcoQO |
| Seals_SH | Trend Grey seals, Schleswig-Holstein | EcoQO |
| TN_LS | Total nitrogen, Lower Saxony, standardized to 30 psu | EcoQO |
| TN_SH | Total nitrogen, Schleswig-Holstein, stand. to 30 psu | EcoQO |
| TP_LS | Total phosphorus, Lower Saxony, stand. to 30 psu | EcoQO |
| TP_SH | Total phosphorus, Schleswig-Holstein, stand. to 30 psu | EcoQO |
| VT_Festrub | Trend vegetation cover Festuca rubra saltmarsh, Hamburger Hallig | Ecosystem parameter |
| VT_Halport | Trend vegetation cover Halimione portulacoides saltmarsh, Hamburger Hallig | Ecosystem parameter |
| VT_LowMarsh | Trend vegetation cover Spartina spp. saltmarsh, Hamburger Hallig | Ecosystem parameter |
| VT_Pucmar | Trend vegetation cover Puccinellia maritima saltmarsh, Hamburger Hallig | Ecosystem parameter |
| WBM_LS | Benthic winter biomass Lower Saxony , Wadden Sea (Norderney) | Ecosystem parameter |
| WBM_NLD | Benthic winter biomass , Dutch Wadden Sea (Scheveplat) | Ecosystem parameter |
| f284 | Trend Grey gurnard, southern North Sea, IBTS Q3 | Ecosystem parameter |
| f293 | Trend cod, southern North Sea, IBTS Q3 | EcoQO |
| f407 | Trend dab, southern North Sea, IBTS Q3 | Ecosystem parameter |
| f465 | Trend whiting, southern North Sea, IBTS Q3 | Ecosystem parameter |
| f471 | Trend hake, southern North Sea, IBTS Q3 | EcoQO |
| f607 | Trend plaice, southern North Sea, IBTS Q3 | EcoQO |
| f639 | Trend Thornback ray, southern North Sea, IBTS Q3 | Ecosystem parameter |
| f735 | Trend Spiny dogfish, southern North Sea, IBTS Q3 | Ecosystem parameter |
| f716 | Trend sole, southern North Sea, IBTS Q3 | EcoQO |
| PHI40 | PHI40 indicator occurrence large fish, southern North Sea, IBTS Q3 | Ecosystem parameter |
| CT_sumDDTtrischen | DDT in eggs Common tern, Trischen | EcoQO |

#### Table S1App.2 : R code for worked example

| library(lattice)  library(reshape2)  library(sampling)  library(vegan)  library(ggplot2)  library(sads)  library(stringr)  library(tigerstats)  # Loading data  all.data<-read.table("Worked example Fock Kraus.csv", header = TRUE, sep = ";", quote = "\"", dec = ".",  fill = TRUE, comment.char = "")  # removing YEAR and selecting complete cases  correct<-all.data[,-c(1)]  correct<- correct[complete.cases(correct),]  all.data_pca<-prcomp(correct, scale=TRUE)  par(mar=c(3,3,3,3))  tiff("Plot_box1.tiff", height = 6, width = 6, units = 'in', type="windows", compression="lzw+p", res=300)  biplot(all.data_pca)  dev.off()  summary(all.data_pca)  # setting up rda with 48 % explained by "expl" variables  expl<-correct[, c(9, 13)] # expanatory variables  resp<-correct #response variables including all  myrda1<-rda(resp, expl, scale=TRUE)  summary(myrda1)  # selecting those with reference values, 42 % explained by LFI and CT_ppDDE (Common tern)  expl<-correct[, c(7, 9)]  resp<-correct  myrda2<-rda(resp, expl, scale=TRUE)  summary(myrda2)  # Likelihoods berechnen  attach(correct)  target.DDT<-10  target.LFI<-0.3  sd.LFI<-0.035*sqrt(50)/1.96 # taken from CI's  sd.ppDDE<-sd(CT_ppDDE)  ref.ppDDE<-round(825/113*target.DDT,2) # recalculated based on mammal LC50 DDT=113 DDE=825  # LFI 2007 = 0.129, LFI 2008 = 0.135, ppDDE 2007 = 170, ppDDE 2008 = 90  LFI07<-0.29  LFI08<-0.29  ppDDE07<-11  ppDDE08<-11  L.07prior<- 0.55  # Probabilities L for GES=y(es) and GES=n(o)  Ly.LFI.08<-pnormGC(0.3, region="above", mean=LFI08, sd=sd.LFI , graph=TRUE) # check with graph  Ly.LFI.07<-pnormGC(0.3, region="above", mean=LFI07, sd=sd.LFI , graph=TRUE)  Ly.ppDDE07<-pnormGC(ref.ppDDE, region="below", mean=ppDDE07, sd=sd.ppDDE , graph=TRUE)  Ly.ppDDE08<-pnormGC(ref.ppDDE, region="below", mean=ppDDE08, sd=sd.ppDDE , graph=TRUE)  Ln.LFI.08<-pnormGC(0.3, region="below", mean=LFI08 , sd=sd.LFI , graph=TRUE)  Ln.LFI.07<-pnormGC(0.3, region="below", mean=LFI07 , sd=sd.LFI , graph=TRUE)  Ln.ppDDE07<-pnormGC(ref.ppDDE, region="above", mean=ppDDE07 , sd=sd.ppDDE , graph=TRUE)  Ln.ppDDE08<-pnormGC(ref.ppDDE, region="above", mean=ppDDE08 , sd=sd.ppDDE , graph=TRUE)  # Probability 2007  L.07<- (Ly.ppDDE07*Ly.LFI.07)*L.07prior/((Ln.ppDDE07*Ln.LFI.07)*(1-L.07prior)+(Ly.ppDDE07*Ly.LFI.07)*(L.07prior))  # Probability 2008  L.08<- (Ly.ppDDE08*Ly.LFI.08)*L.07/((Ln.ppDDE08*Ln.LFI.08)*(1-L.07)+(Ly.ppDDE08*Ly.LFI.08)*(L.07)) |
| --- |

#### Fig. S1App.1 : PCA biplot of analysis according to code in Table S4.2


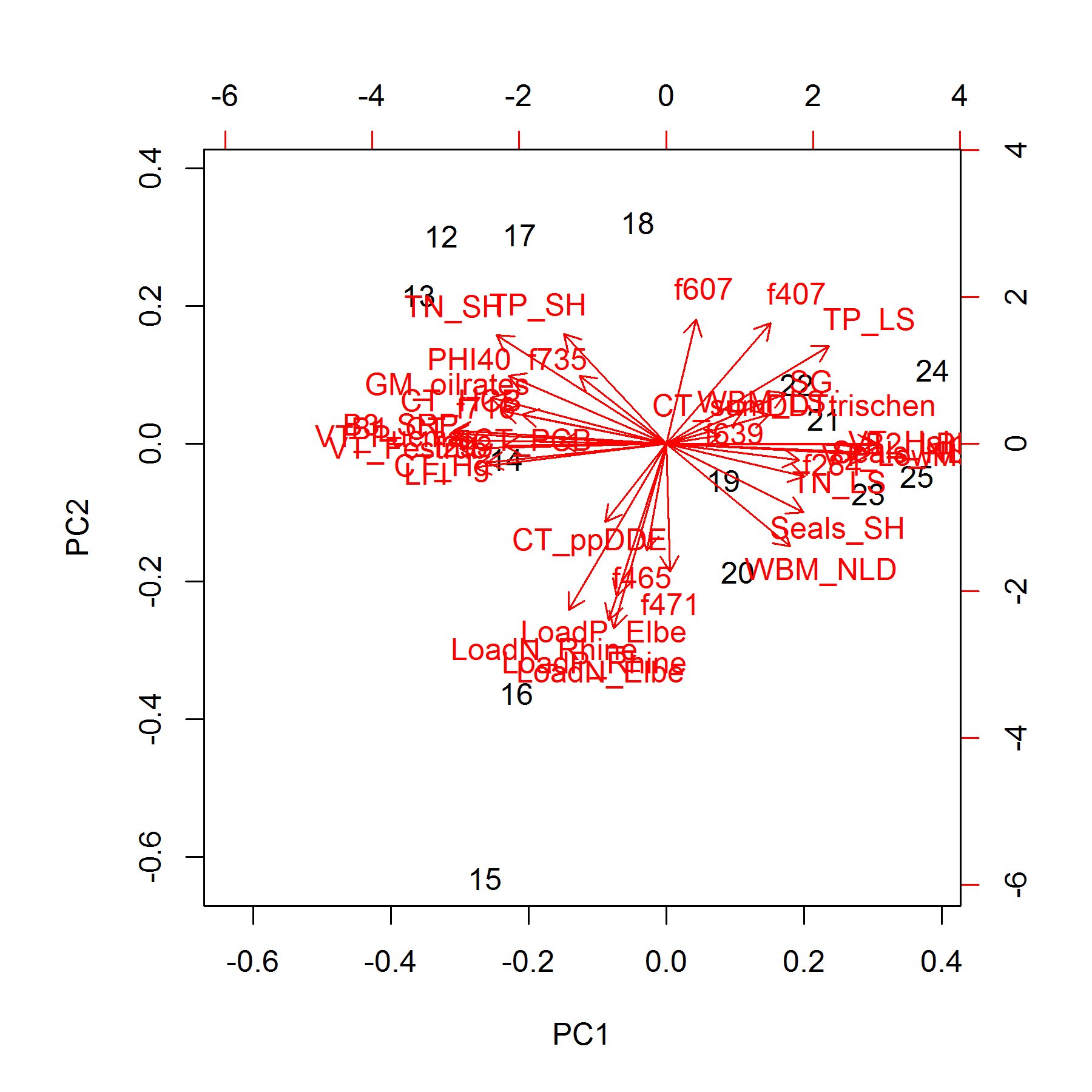

Supplement: S1 Appendix — (2 files) Worked examples of an assessment of Good Environmental Status (GES) in the ex-post branch in relation to (a) a theoretical case study focusing on one-out-all-out (OOAO) and the heuristic approach (HA) and (b) an application of HA to OSPAR data and EcoQOs for the Southern North Sea (ZIP) [file pone.0159481.s001.zip › Fock Kraus S1 Appendix.docx]
