# Supplementary material for: From Metaphors to Formalism: A Heuristic Approach to Holistic Assessments of Ecosystem Health
Source: PLoS One. 2016 Aug 10;11(8):e0159481. doi: 10.1371/journal.pone.0159481 (PMC4980027; doi:10.1371/journal.pone.0159481)
Supplement: S1 Table — As indicated in European Commission Directive 2008/56/EC Annex I. (DOCX) [file pone.0159481.s002.docx]

S1 Table

Supporting information to

From metaphors to formalism: A heuristic approach to holistic assessments of ecosystem health

Heino O. Fock, Gerd Kraus

[S1 Table: MSFD qualitative descriptors for determining good environmental status 2](#_Toc457289449)

## S1 Table: MSFD qualitative descriptors for determining good environmental status

As indicated in European Commission Directive 2008/56/EC Annex I

(1) Biological diversity is maintained. The quality and occurrence of habitats and the distribution and abundance of species are in line with prevailing physiographic, geographic and climatic conditions.

(2) Non-indigenous species introduced by human activities are at levels that do not adversely alter the ecosystems.

(3) Populations of all commercially exploited fish and shellfish are within safe biological limits, exhibiting a population age and size distribution that is indicative of a healthy stock.

(4) All elements of the marine food webs, to the extent that they are known, occur at normal abundance and diversity and levels capable of ensuring the long-term abundance of the species and the retention of their full reproductive capacity.

(5) Human-induced eutrophication is minimized, especially adverse effects thereof, such as losses in biodiversity, ecosystem degradation, harmful algae blooms and oxygen deficiency in bottom waters.

(6) Sea-floor integrity is at a level that ensures that the structure and functions of the ecosystems are safeguarded and benthic ecosystems, in particular, are not adversely affected.

(7) Permanent alteration of hydrographical conditions does not adversely affect marine ecosystems.

(8) Concentrations of contaminants are at levels not giving rise to pollution effects.

(9) Contaminants in fish and other seafood for human consumption do not exceed levels established by Community legislation or other relevant standards.

(10) Properties and quantities of marine litter do not cause harm to the coastal and marine environment.

(11) Introduction of energy, including underwater noise, is at levels that do not adversely affect the marine environment.
