# Supplementary material for: From Metaphors to Formalism: A Heuristic Approach to Holistic Assessments of Ecosystem Health
Source: PLoS One. 2016 Aug 10;11(8):e0159481. doi: 10.1371/journal.pone.0159481 (PMC4980027; doi:10.1371/journal.pone.0159481)
Supplement: S2 Table — (DOCX) [file pone.0159481.s003.docx]

S2 Table

Supporting information to

From metaphors to formalism: A heuristic approach to holistic assessments of ecosystem health

Heino O. Fock, Gerd Kraus

[S2 Table: Approaches proposed for the MSFD for aggregation of different metrics, indicators or criteria to assess good environmental status, including the advantages and disadvantages of each approach, covering modeling and indicator based methods. 2](#_Toc457289757)

[S2.1 - Indicator based / ex-post assessments 2](#_Toc457289758)

[S2.2 -Model based assessments 6](#_Toc457289759)

[References 7](#_Toc457289760)

## S2 Table: Approaches proposed for the MSFD for aggregation of different metrics, indicators or criteria to assess good environmental status, including the advantages and disadvantages of each approach, covering modeling and indicator based methods.

### S2.1 - Indicator based / ex-post assessments

Two methods mentioned by reports are excluded, i.e. ‘checklist approach’ and ‘combination of methods’ ([Borja et al., 2013](#_ENREF_5), [Borja et al., 2014](#_ENREF_8)), since no specific algorithm is included in their rationale. As such, the rule based method of Shepherd et al. [1] and the combination of OOAO with probability distributions in Probst et al. [2] were counted as ‘combination of methods’. Together with the latter, Greenstreet et al. [3] proposed the use of tests based on probability distributions, which due to a lack of conditional independence appears incorrect, since correlated time series subject to the same management and environmental regime cannot be regarded as independent draws from a random distribution of events. Sources include mainly review papers containing many of the original works referred to therein.

| **Name** | **Nr** | **Assessment type** | **Model type** | **Information retainment** | **Details of**  **Method** | **Advantages as indicated by source** | **Disadvantages as indicated by source** | **Source** | **Comments** |
| --- | --- | --- | --- | --- | --- | --- | --- | --- | --- |
| One-out all-out  (OOAO) principle | 1 | Pressure indicator based | Quantitative | Exclusive | All variables have to  achieve good status. | Most comprehensive  approach. Follows the  precautionary principle. | Trends in quality are  hard to measure. Chance of failing to achieve good status very high. May include double-counting. | [4] | Biased due to type II error properties. Mainly suggested for pressure indicators. |
| Two-out all-out | 2 | Pressure indicator based | Quantitative | Exclusive | if two  variables do not meet the  required standard, good  status is not achieved | More robust compared to  OOAO approach | See above | [5] | Marginally improved type II error properties, still not recommendable Two-out-all-out is also considered conditional rule (see Nr. 3). Mainly suggested for pressure indicators. |
| Conditional rules | 3 | Pressure/state indicator based | Quantitative | Exclusive | A specific proportion of the  variables have to achieve  good status | Can help to focus on the key aspects | Assumes that GES is well represented by a selection of variables. Relies on expert judgement and OOAO, see Breen et al. [6] | [5] | Applying OOAO this method underrates GES. Expert judgment requires extensive consideration of uncertainty [7] |
| High-level  integration | 4 | Pressure/state indicator based | Quantitative | Exclusive | Assessment results for  some pre-defined groups  each  applying OOAO | Reduces the risks  associated with OOAO  while still giving an overall  assessment  Reduced number of indicators | Technical details | [5] | Applying OOAO this method underrates GES. Expert judgment requires extensive consideration of uncertainty [7] |
| Averaging / summing approach | 5 | Pressure/state indicator based | Quantitative | Additive | Non-weighted: Variable values are combined, using the arith-metic average or median | Indicator values can be calculated at each level of aggregation.  Recommended when  Combined parameters are sensitive to a single pressure | Assumes all variables  are of equal impor-tance. Assigning weights by expert judgment introduces bias. | [8] | Weighting does not account for conditional independence. An aspect of arbitrariness remains in all protocols where weighting and scoring is applied [7–9] |
| Averaging / summing with weights | 6 | Pressure/state indicator based | Quantitative | Additive | Weighted: Like the  previous method, with  different weights assigned  to  the various variables | Reflects the links between descriptors and avoids double counting | High data requirements  Problem of agreeing on weights | [8,10,11] | Weighting does not account for conditional independence. See comment on weighting in #5. |
| Averaging with hierarchy | 7 | Pressure/state indicator based | Quantitative | Additive | Hierarchical: With  variables defined at  different hierarchical  levels | Reflects the hierarchy  among descriptors and  avoids double counting, Different calculation rules  can be applied at different  levels | Problem of agreeing on hierarchy, hierarchy influences assessment | [8] | Weighting does not account for conditional independence. See comment on weighting in #5. |
| Scoring or rating | 8 | Pressure/state indicator based | Quantitative | Additive | Sum of weighted  scores | Different weights can be  assigned to the various  elements | Problem of agreeing on weights which must be on cardinal scale [4], otherwise the sum of scores is not defined mathema-tically. Metrics may not be sensitive to the same Pressures | [5,8] | Scoring does not account for conditional independence. |
| State space | 9 | State indicator based | Quantitative | Additive | Develop trajectory of state variables in relation reference condition | Accounts for ecosystem variability | Reference condition arbitrary | [12] | The state space model and the Bayesian approach account for variability of ecosystem state in a certain domain. |
| Multi-metric  approaches | 10 | State indicator based | Quantitative | Additive | Multi-metric indices inte-grate multiple indicators into one value. | May result in  more robust  indicators, compared  to indicators based on  single parameters | Correlations between  parameters are an issue (redundancy problem) [13]. Results are hard to communicate to managers. Metrics may not be sensitive to the same pressures | New synthesis indicator  [5,8]; for D6 see OSPAR [14] and review by ICES [15] | Complies with additive utility model, based on conditionally independent indicators. The problem of redundant information is known but not solved. |
| Multi-dimensional  approaches | 11 | State indicator based | Quantitative | Additive | Multivariate analyses | No need to set rigid target values, since values are  represented within a domain, i.e. need to vary | Results are hard to  communicate to  managers | [8] | Condionally independent. Interpreation of surrogate indicators is criticial but still a feasible mthod covering a high amount of ecosystem variability. |
| Probabilistic | 12 | State indicator based | Quantitative | Additive | Likelihoods and log likelihoods can be displayed, coherent rules to calculate final score | Accounts for uncertainty in data, allows to include different types of information. |  | [5] | Conditional independence issue not considered in source papers for likelihood functions. Conditional independence required to understand Bayesian reasoning |
| Spider web | 13 | Pressure/state indicator based | Quantitative | Additive | The shape of the diagram | Easy to under-stand and show to managers | The decision on when GES is  achieved | [4] |  |
| Decision tree | 14 | Pressure/state indicator based | Quantitative | Additive | Integrating elements into a  quality assess-ment using  specific decision  rules | Possible to combine  different types of  elements, flexible  approach | Only quantitative up to a certain level | [5] |  |
| Pressures only | 15 | Pressure indicator based | Quantitative | Additive | No pressures in an area sufficient to  cause adverse effects | Can be derived by national  databases, map-ping, pressure  lists | Relates to ‘cause’ rather than  ‘effect’, difficult to set boundaries between pressure  status classes: is it sufficient to  base the assess-ment on the list of pres-sures, while those can have very different spatial extent and strength? | [4] | Relates to risk assessments and can be conducted in the absence of operational state indicators, but is insufficient in that only GES proxy can be derived - see assessment framework #11 for the consideration of resistance and resilience in that respect. |
| Biodiversity only | 16 | State indicator based | Quantitative | Additive | All biodiversity  indicators are met irrespective of weighting | Focuses on the  main aspect | Assumes that the biodiversity  descriptor really does encompass all others | [4] | Not all indicators for instance from the OSPAR EcoQO list are biodiversity indicators, but biodiversity indicators will be core indicators for indicator based assessments since they directly link to GES – see assessment framework #11 for the consideration of resistance and resilience in that respect |
| Connectivity Matrix | 17 | Pressure indicator based | Qualitative | Additive | Linking pressures and ecosystem components | Easy to identify important pressures | Measuring presence / absence of pressures and thus incapable of measuring incre-mental change and impact | [16] |  |

### S2.2 -Model based assessments

In the context of the DEVOTES project it was shown that whole ecosystem models are highly relevant if they include links to biodiversity components ([Smith et al., 2014](#_ENREF_32)).

| **General approach** | **Nr** | **Assessment type** | **Model type** | **Information treatment** | **Details of**  **Method** | **Advantages** | **Disadvantages** | **Source** |
| --- | --- | --- | --- | --- | --- | --- | --- | --- |
| Risk models | 18 | Model based | Quantitative | Focusing on DPSIR chains |  | Risk models can be applied in high spatial resolution and facilitate easy target setting. Upside (gain) and downside risk (loss) can be applied, so that a gain can serve as proxy for GES | The risk concept is often misperceived as probability. | [17] |
| Dynamic models | 19 | Model based | Quantitative | Ecosystem models |  | Can test the robustness of simpler models ([Hilborn, 2003](#_ENREF_18)) | Data hungry models, e.g. ECOPATH with ECOSIM, ATLANTIS or coupled lower trophic and high trophic models (see DEVOTES Deliverable 4.1. Report on available models for biodiversity and needs for development). | [18] |

## References

1. Shephard S, Rindorf A, Dickey-Collas M, Hintzen NT, Farnsworth K, Reid DC. Assessing the state of pelagic fish communities within an ecosystem approach and the European Marine Strategy Framework Directive. ICES J Mar Sci. 2014;doi:10.109: 1–14.

2. Probst WN, Kloppmann M, Kraus G. Indicator-based status assessment of commercial fish species in the North Sea according to the EU Marine Strategy Framework Directive (MSFD). ICES J Mar Sci. 2013; doi.10.1093/icesjms/fst010.

3. Greenstreet SPR, Rossberg AG, Fox CJ, Le Quensne WJF, Blasdale T, Boulcott P, et al. Demersal fish biodiversity: species-level indicators and trends-based targets for the Marine Strategy Framework Directive. ICES J Mar Sci. 2012;69: 1789–1801.

4. Borja A, Elliott M, Andersen JH, Cardoso AC, Carstensen J, Ferreira JG, et al. Good Environmental Status of marine ecosystems: What is it and how do we know when we have attained it? Mar Pollut Bull. 2013;76: 16–27.

5. Borja A, Prins T, Simboura N, Andersen JH, Berg T, Marques JC, et al. Tales from a thousand and one ways to integrate marine ecosystem components when assessing the environmental status. Front Mar Sci. 2014;1:22. doi:doi: 10.3389/fmars.2014.00022

6. Breen P, Robinson LA, Rogers SI, Knights AM, Piet G, Churlova T, et al. An environmental assessment of risk in achieving good environmental status to support regional prioritisation of management in Europe. Mar Policy. 2013;36: 1033–1043.

7. Rochet M-J, Rice JC. Do explicit criteria help in selecting indicators for ecosystem-based fisheries management? ICES J Mar Sci. 2005;62: 528–539.

8. Prins T, Meulen M van der, Boon A, Simboura N, Tsangaris C, Borja A, et al. Coherent geographic scales and aggregation rules for environmental status assessment within the Marine Strategy Framework Directive. Deltares/AZTI/HCMR; 2014.

9. Hayashi K. Multicriteria analysis for agricultural resource management: A critical survey and future perspectives. Eur J Oper Reserach. 2000;122: 486–500.

10. Borja A, Elliott M, Carstensen J, Heiskanen A-S, van de Bund W. Marine management - Towards an integrated implementation of the European Marine Strategy and the Water Framework Directives. Mar Pollut Bull. 2010;60: 2175–2186.

11. Borja A, Galparsoro I, Irigoien X, Iriondo A, Menchaca I, Muxika I, et al. Implementation of the Europen Marine Strategy Framework Directive: a methodological approach for the assessment of environmental status, from the Basque Country (Bay of Biscay). Mar Pollut Bull. 2011;62: 889–904.

12. Tett P, Gowen RJ, Painting SJ, Elliott M, Forster R, Mills DK, et al. Framework for understanding marine ecosystem health. Mar Ecol Progess Ser. 2013;494: 1–27.

13. Primpas I, Karydis M. Scaling the trophic index (TRIX) in oligotrophic marine environments. Environ Monit Assess. 2011;178: 257–269.

14. OSPAR Commission. Report of the OSPAR workshop on MSFD biodiversity descriptors: comparison of targets and associated indicators [Internet]. Biodiversity Series. 2012. Available: http://www.ospar.org/documents/dbase/publications/p00575_ospar biodiversity workshop.pdf

15. ICES. 1.5.6.3 Special request, Advice June 2013, OSPAR special request on review of the technical specification and application of common indicators under D1, D2,, D4, and D6. 2013;

16. Knights AM, Koss RS, Robinson LA. Identifying common pressure pathways from a complex network of human activities to support ecosystem-based management. Ecol Appl. 2013;23: 755–765.

17. Fock HO, Kloppmann M, Stelzenmüller V. Linking marine fisheries to environmental objectives: A case study on seafloor integrity under European maritime policies. Environ Sci Policy. 2011;14: 289–300. doi:10.1016/j.envsci.2010/11.005

18. Smith C, Papadopoulou N, Barnard S, Mazik K, Patrício J, Elliott M, et al. Conceptual models for the effects of marine pressures on biodiversity. Hellenic Centre for Marine Research; 2014.
