# Supplementary material for: From Metaphors to Formalism: A Heuristic Approach to Holistic Assessments of Ecosystem Health
Source: PLoS One. 2016 Aug 10;11(8):e0159481. doi: 10.1371/journal.pone.0159481 (PMC4980027; doi:10.1371/journal.pone.0159481)
Supplement: S3 Table — Screening is undertaken with respect to three procedural steps essential to the heuristic approach. (DOCX) [file pone.0159481.s004.docx]

S3 Table

Supporting information to

From metaphors to formalism: A heuristic approach to holistic assessments of ecosystem health

Heino O. Fock, Gerd Kraus

[S3 Table: Comparative screening procedure for selected maritime environmental ecosystem health assessment methodologies 2](#_Toc457290193)

[References 5](#_Toc457290194)

## S3 Table: Comparative screening procedure for selected maritime environmental ecosystem health assessment methodologies

Screening is undertaken with respect to three procedural steps essential to the heuristic approach.

| Name | Assessment type and description | Ex-ante/ex-post approach | Indicator aggregation and normalization | Sources |
| --- | --- | --- | --- | --- |
| Ocean Health Index | Deconstructive-structural with a priori selection of system components: Ten ocean-related societal goals or ecosystem services are selected covering the ecological, social, and economic dimensions of the ocean:  1) ‘Food Provision’ (‘Wild Caught Fisheries’ and ‘Mariculture’),  2) ‘Artisanal Fishing Opportunities’,  3) ‘Natural Products’,  4) ‘Carbon Storage’,  5) ‘Coastal Protection’,  6) ‘Tourism&Recreation’  7) ‘Coastal Liveli- hoods&Economics’ (‘Livelihoods’ and ‘Economics’),  8) ‘Sense of Place’ (‘Iconic Species’ and ‘Lasting Special Places’), and  9) ‘Clean Waters’, and  10) ‘Biodiversity’ (‘Species’ and ‘Habitats’), | Mixed approach: The calculation step includes modeling on present and future information (trends), pressures on this goal and the recovery potential. All goals are substitutable with each other.  The index includes present value and near-future trends, therefore is assigned to period rather than point in time. The inclusion of future pressures allows to test management scenarios, but also introduces uncertainty given that the perception of future trends may differ between expert judgments, example North Sea trends in pressures and status of maritime habitats in [1,2]. | Additive model with between domain normalization: A global and local index is calculated by taking the weighted arithmetical average score of these goals after normalized to reference value (Ecological Quality Ratios , EQR). Weighting and selection criteria based on expert judgment. | Main reference: [2]  Review: [3] |
| Baltic Sea Holistic Assessment (HOLAS) | Deconstructive-structural with a priori selection of system components: From a set of predefined indicators from the CORESET program 3 thematic assessments are undertaken: The HELCOM Eutrophication Assessment Tool (HEAT), the HELCOM Biodiversity Assessment Tool (BEAT), based on HEAT on HEAT methodology, the HELCOM Hazardous Substances Status Assessment Tool (CHASE). The three thematic assessments are accompanied by the Baltic Sea Pressure Index (BSPI). | Ex-post thematic assessments based on indiocators. Ex-ante branch as impact assessment BSPI which follows Halpern et al. [4], but has the problem of boundary setting including how to determine what is good environmental status. HOLAS is under revision. | Mixed additive-exclusive aggregation with between-domain normalization:  Within indicator groups additive aggregation is applied as weighted arithmetic mean, weighting is based on expert judgment. To aggregate groups, OOAO is applied.  Values are used as Ecological Quality Ratios EQR, normalized to reference value [5].  For HOLAS 1, the unweighted arithmetic mean is taken for the three thematic assessments to generate HOALS as holistic assessment, but the ex-ante branch is not integrated. | Main reference: [5–8]  Review: [9] |
| EU Water Framework Directive assessments (WFD) | Deconstructive-structural with a priori selection of system components: WFD establishes a framework for the protection of groundwater, inland surface waters, rivers, lakes, and coastal inshore waters, based on quality elements defined in Annex V. | Ex-post: For each of the water bodies, biological, hydromorphological and physico-chemical ‘quality elements’ are defined in Annex V, together with normative definitions on high, good and moderate status. | Exclusive: The assessment method for the quality elements is one-out-all-out, based on 5 quality levels for each element, see WFD Annex V 1.4.2. Quality levels are determined from Ecological Quality Ratios (EQR). | Main reference: European Commission (2000/60/EC)  Review: [10,11] |
| OSPAR Quality Status Reports | Deconstructive-structural with a priori selection of system components: The OSPAR quality Status Report (QSR) consists of thematic reports and overall evaluation. This is accompanied by the ecosystem assessment, for which 9 ecological quality issues are described in terms of 11 established ecological quality objectives (EcoQo). EcoQOs are :  • Annual by-catch of harbour porpoises  • Seal Population Trends  • (Commercial fish species)  • Large fish indicator  • Oxygen deficiency  • Primary production / eutrophication  • Mercury concentrations in bird eggs in industrialized estuaries  • Concentrations of organochlorines in seabird eggs  • Proportion of oiled seabirds  • Benthic imposex  • Plastic particles in seabird stomachs | Ex-post approach: Hazardous substances are evaluated against background concentration values and assessed as unacceptable, acceptable or background/zero concentration without further scaling or normalizing. For EcoQIs, respective EcoQOs have defined targets based on trend or abundance values.  OSPAR EcoQOs have played an important role in developing MSFD descriptors [12]. | Qualitative without normalization: OSPAR applies a so-called qualitative assessment in the 2010 QSR. Each thematic assessment is summarized in text form. The ‘Utrecht assessment methodology’ mainly applied one-out-all-out criteria in terms of worst case scenarios in addition to the were applied to the general assessment. The general assessment comprised categorical analysis based on expert judgment for weighting and scoring of impacts, magnitude and trend, the ‘Utrecht procedure in considered for revision. | Main reference: [1]  Review: [12–14] |

## References

1. OSPAR Commission. Quality Status report 2010. London: OSPAR Commission; 2010.

2. Halpern BS, Longo C, Hardy D, McLeod KL, Samhouri JF, Katona SK, et al. An index to assess the health and benefits of the global ocean. Nature. 2012;488: 615–622.

3. Rickels W, Quaas MF, Visbeck M. How healthy is the human-ocean system? Enviromental Reserach Lett. 2014;9: 15.

4. Halpern BS, Walbridge S, Selkoe KA, Kappel C V, Micheli F, D’Agrosa C, et al. A Global Map of Human Impact on Marine Ecosystems. Science (80- ). 2008;319: 948–952.

5. Andersen JH, Dahl K, Göke C, Rindorf A, Skov H, Vinther M, et al. Integrated assessment of marine biodiversity status using a prototype indicator-based assessment tool. Front Mar Sci. 2015;

6. HELCOM. Final draft project description for HELCOM HOLAS II. 2014. Report No.: HOD 46-2014.

7. HELCOM. Towards a tool for quantifying anthropogenic pressures and potential impacts on the Baltic Sea marine environment [Internet]. Helsinki, Finland; Baltic Sea Environmental Proceedings 125, 2010. p. 69. Available: http://helcom.fi/Lists/Publications/BSEP125.pdf

8. HELCOM. Ecosystem Health of the Baltic Sea 2003-2007: HELCOM Initial Holistic Assessment [Internet]. Helsinki: HELCOM; 2010. Available: http://www.helcom.fi/stc/files/Publications/Proceedings/bsep122.pdf

9. Piha H, Zampoukas N. Review of Methodological Standards Related to the maritime Strategy Framework Directive Criteria on Godd Environmental Status. JRC Scientific and Technical Reports. JRC; 2011.

10. Hatton-Ellis T. The Hitchhiker’s Guide to the Water Framework Directive. Aquat Conserv Mar Freswater Ecosyst. 2008;18: 111–116.

11. Borja A, Rodriguez JG. Problems associated with the “one-out, all-out” principle, when using multiple ecosystem components in assessing the ecological status of marine waters. Mar Pollut Bull. 2010;60: 1143–1146.

12. Heslenfeld P, Enserink EL. OSPAR Ecological Quality Objectives: the utility of health indicators for the North Sea. ICES J Mar Sci. 2008;65: 1392–1397.

13. OSPAR Commission. Evaluation of the OSPAR system of Ecological Quality Objectives for the North Sea (update 2010). 2010.

14. Johnson D. Environmental indicators: their utility in meeting the OSPAR Convention’s regulatory needs. ICES J Mar Sci. 2008;65: 1387–1391.
