# Supplementary material for: From Metaphors to Formalism: A Heuristic Approach to Holistic Assessments of Ecosystem Health
Source: PLoS One. 2016 Aug 10;11(8):e0159481. doi: 10.1371/journal.pone.0159481 (PMC4980027; doi:10.1371/journal.pone.0159481)
Supplement: S1 Text — (DOCX) [file pone.0159481.s005.docx]

S1 Text

From metaphors to formalism: A heuristic approach to holistic assessments of ecosystem health

Heino O. Fock, Gerd Kraus

[S1 Text. MFSD assessment framework 1](#_Toc457289638)

[Needs for the MSFD 2](#_Toc457289639)

[References 3](#_Toc457289640)

## S1 Text. MFSD assessment framework

MSFD defines two fields of activity, i.e. the assessment of GES carried out periodically every six years, and the implementation of measures to achieve this aim, i.e. phasing-out of pressures (MSFD Article 1). Eleven descriptors of GES (S2 Table) are characterized by a list of physical, chemical and biological attributes, pressures and impacts. The primary list of descriptors from 2008 did not resolve the ex-ante/ ex-post dichotomy essential for adaptive management designs (Mee et al., 2008). However, a *post hoc* classification into pressure and state descriptors was undertaken after 2008 (Claussen et al., 2011), however still without specifying pressure-state-response chains (PSR) as state-of-the-art in European integrative assessments (Johnson, 2008). In 2010, an additional list of standards was published by the European Commission (2010/477/EU), specifying 56 generic indicators and a hierarchical aggregation framework to assign indicators to criteria and criteria to descriptors. Links between indicators were not specified. It may be expected that when the existing generic indicators (e.g. ‘fishing mortality’) are specified (e.g. ‘fishing mortality for cod in the North Sea’), 56+X need to be assessed in practice. The DEVOTES project identified 557 indicators (Patrício et al., 2014). The tendency to increase the number of indicators beyond 56+X may be counterbalanced by an upcoming revision of Commission Decision 2010/477/EU (e.g. ICES, 2014).

### Needs for the MSFD

The identification of MFSD needs follows the legal text (Table 1) and the first revisions of the implementation of the MSFD (e.g. COM(2010/477/EU)). Gaps remain regarding socio-economic aspects, undefined PSR chains, undefined targets for indicators, the meaning of GES at system level and appropriate aggregation and assessment tools (Bertram et al., 2014; Borja et al., 2014; CPMR, 2014; Prins et al., 2014). Nineteen assessment methods have been proposed to date for MSFD assessments (S3 Table). Mainly mixed concepts are advocated, with exclusive aggregation for pressure and additive aggregation for state indicators but without guidance how to achieve the overall assessment. The suggested method for the exclusive assessment is one-out-all-out (OOAO), which is considered the decision making tool for the precautionary principle (PP) (Hatton-Ellis, 2008; Prins et al., 2014; Tett et al., 2013). In OOAO, the worst status of all assessed elements determines the assessment of overall performance (Borja and Rodriguez, 2010). Element-wise evaluations in OOAO make it easy to aggregate data, but it proves to be trend insensitive (Prins et al., 2014) and type II error features render OOAO unmanageable in practical terms (Borja et al., 2013).

From a management perspective a more practical assessment of GES, compliance with the precautionary principle and the ecosystem-based approach to management, avoidance of redundancy between indicators, differential approaches for pressure and state descriptors, methodologies to aggregate information between descriptors, and methods for a flexible reporting on the status beyond OOAO are needed (Claussen et al., 2011).

Table 1. Comparison of current assessment approaches and heuristic approach with reference to MSFD legal text. Where no guidance is available from the legal text (NA), reference is given to reports from EU working groups.

| Criterion | Reference to MSFD text | Current suggested MSFD assessment methodology state 2014 | Heuristic approach, this paper |
| --- | --- | --- | --- |
| Normative dimension: Aim | Art. 1(1) , Art 3(5) | Ecosystem health / GES | Ecosystem health in a multidimensional sustainability context |
| Normative dimension: Multidimensionality | Art. 7(1) | Undefined, but analysis of uses and cost-of-degradation analysis requested | Evaluation of scenarios in ex-ante assessments which can be based on socio-economic sustainability criteria |
| Normative dimension: Types of GES indices | Art. 3(5) | One holistic measure of GES but undefined | One holistic measure of GES plus several model based relativistic measures to develop phasing-out of pressures |
| Normative dimension: Assessment type | Art. 1(1) with 3(8), Art. 10 | Ex-post approach distinguishing between state/pressure indicators but undefined, no ex-ante assessment defined | Dual concept with separated ex-ante/ex-post approach with conjunction to evaluate resilience and provide guidance on phasing-out strategy |
| Normative dimension: Index development | NA | Undefined, but aiming at aggregating across the attribute-indicator-criterion-descriptor MSFD hierarchy (see (2010/477/EU)) | Additive in ex-post, development of causal PSR networks in ex-ante section. MSFD attribute-indicator-criterion-descriptor hierarchy retained in analysis of indicator space |
| Normative dimension: GES measurement scale | NA | Binary: GES/non-GES (Claussen et al., 2011) | Gradual in ex-post assessment: GES probability scale |
| Normative dimension: Flexibility and adaptive potential | Art. 3(5) | Undefined | Adaptive through combination of ex-post and ex-ante assessments |
| Normative dimension: Precautionary principle retained | Preambles 27, 44 | …in assessment methodology OOAO – high type II error properties | … in target setting in ex-post assessments |
| Normative dimension: Ecosystem-based approach | Preamble 44, Art. 8 | Extensive list of attributes in MSFD Annex 3 plus indicator/descriptor hierarchy | *A* *posteriori* analysis of ecosystem variability in indicator space^a^ |
| Systemic dimension: Number of indicators and PSR chains | Annex I, concluding remark | 56+X / no guidance on PSR chains (see (2010/477/EU)) | Selection of independent indicators and PSR chains after analysis of indicator space and pressure space. |
| Normative dimension: Normalization of data | NA | Cross domain normalization retaining the apples and oranges-problem (Prins et al., 2014) | Within domain normalization both in ex-ante assessment e.g. risk models, and ex-post-assessment, here Bayesian approach with likelihood functions |
| Normative dimension: Aggregation procedure | NA | Preference for combined additive and exclusive modeling, unjustified combination of both (Prins et al., 2014) | Ex-post: Additive utility function based on likelihoods models within a Bayesian framework; ex-ante: risk models and others |

^a^ The analysis of indicator space and subsequent selection of indicators implies a re-interpretation of the terms ‘essential features’ (=selected variables) and ‘indicative list of elements’ (=indicator space) of MSFD Article 8, which states the obligation to analyze “the essential features and characteristics, …, based on the indicative lists of elements set out in Table 1 of Annex III”.

## References

Bertram, C., Dworak, T., Görlitz, S., Interwies, E., Rehdanz, K., 2014. Cost-benefit analysis in the context of the EU Marine Strategy Framework Directive: The case of Germany. Mar. Policy 43, 307–312.

Borja, A., Elliott, M., Andersen, J.H., Cardoso, A.C., Carstensen, J., Ferreira, J.G., Heiskanen, A.-S., Marques, J.C., Neto, J.M., Teixeira, H., Uusitalo, L., Uyarra, M.C., Zampoukas, N., 2013. Good Environmental Status of marine ecosystems: What is it and how do we know when we have attained it? Mar. Pollut. Bull. 76, 16–27.

Borja, A., Prins, T., Simboura, N., Andersen, J.H., Berg, T., Marques, J.C., Neto, J.M., Papadopoulou, N., Reker, J., Teixeira, H., Uusitalo, L., 2014. Tales from a thousand and one ways to integrate marine ecosystem components when assessing the environmental status. Front. Mar. Sci. 1:22. doi:10.3389/fmars.2014.00022

Borja, A., Rodriguez, J.G., 2010. Problems associated with the “one-out, all-out” principle, when using multiple ecosystem components in assessing the ecological status of marine waters. Mar. Pollut. Bull. 60, 1143–1146.

Claussen, U., Connor, D., Vrees, L. de, Leppänen, J.-M., Percelay, J., Kapari, M., Mihail, O., Ejdung, G., Rendell, J., 2011. Common Understanding of (Initial) Assessment, Determination of Good Environmental Status (GES) & Establishment of Environmental Targets (Articles 8, 9 & 10 MSFD).

CPMR, 2014. Technical Paper from the CPMR General Secretariat - Marine Environment and Develepment of the regions: Issues raised by Maritime Strategy Framework Directive (MSFD). Rennes.

Hatton-Ellis, T., 2008. The Hitchhiker’s Guide to the Water Framework Directive. Aquat. Conserv. Mar. Freswater Ecosyst. 18, 111–116.

ICES, 2014. Report of the Workshop to review the 2010 Commission Decision on criteria and methodological standards on good environmental status (GES) of marine waters; Descriptor 6.

Johnson, D., 2008. Environmental indicators: their utility in meeting the OSPAR Convention’s regulatory needs. ICES J. Mar. Sci. 65, 1387–1391.

Mee, L.D., Jefferson, R.L., Laffoley, D. d’A., Elliott, M., 2008. How good is good? Human values and Europe’s proposed Marine Strategy Directive.

Patrício, J., Teixeira, H., Borja, A., Elliott, M., Berg, T., Papadopoulou, N., Smith, C., Luisetti, T., Uusitalo, L., Wilson, C., Krysia Mazik, Niquil, N., Cochrane, S., Andersen, J.H., Boyes, S., Burdon, D., Carugati, L., Danovaro, R., Hoepffner, N., 2014. DEVOTES recommendations for the implementation of the Marine Strategy Framework Directive.

Prins, T., Meulen, M. van der, Boon, A., Simboura, N., Tsangaris, C., Borja, A., Menchaca, I., 2014. Coherent geographic scales and aggregation rules for environmental status assessment within the Marine Strategy Framework Directive. Deltares/AZTI/HCMR.

Tett, P., Gowen, R.J., Painting, S.J., Elliott, M., Forster, R., Mills, D.K., Bresnan, E., Capuzzo, E., Fernandes, T.F., Foden, J., Geider, R.J., Gilpin, L.C., Huxham, M., McQuatters-Gollop, A.L., Malcolm, S.J., Saux-Picart, S., Platt, T., Racault, M.-F., Sathyendranath, S., Molen, J. van der, 2013. Framework for understanding marine ecosystem health. Mar. Ecol. Progess Ser. 494, 1–27.
